# Characterization of some selected macroalgae extracts and assessment of their insecticidal and genotoxicity in *Culex pipiens* L. mosquito larvae

**DOI:** 10.1038/s41598-025-86347-7

**Published:** 2025-01-21

**Authors:** Dina A. Refaay, Mostafa M. El-Sheekh, Yasmin M. Heikal, Ahmed A. Rashed

**Affiliations:** 1 Botany Department, Faculty of Science, Mansoura University, Mansoura, 35516 Egypt; 2 Botany Department, Faculty of Science, Tanta University, Tanta, 31527 Egypt; 3 Economic Entomology Department, Faculty of Agriculture, Mansoura University, Mansoura, 35516 Egypt

**Keywords:** Algal bioinsecticide, Phytochemicals, Larvicidal activity, Comet assay, *Jania rubens*, *Colpomenia sinuosa*, Microbiology, Plant sciences

## Abstract

The continual use of synthetic insecticides to control mosquito larvae has severe implications for human health and the ecosystem, highlighting the need for alternative natural insecticides. Macroalgae may be a good alternative because of their biologically active metabolites with distinctive chemical structures that have been reported for their insecticidal properties. The study aimed to investigate the potential of different extracts from *Jania rubens* (Linnaeus) J.V.Lamouroux and *Colpomenia sinuosa* (Mertens ex Roth) Derbès & Solier as genotoxic and larvicidal agents against *Culex pipiens* Linnaeus larvae. The algae thallus was subjected to extraction using methanol, acetone, and methylene chloride. The phytochemical composition was quantified. The larvicidal activity of the different algae extracts was assessed against the third instar larvae of *Culex pipiens*. Genotoxic evaluation through comet assays and compound characterization by GC/MS was done. The results demonstrated that *J. rubens* methylene chloride extract exhibited the highest contents of phenolics, alkaloids, flavonoids, terpenoids, and tannins while *C. sinuosa* methanol extract demonstrated the highest levels of flavonoids, alkaloids, phenolics, and saponins. The larvicidal activity results revealed that *J. rubens* methylene chloride extract was the most toxic for *C. pipiens* larvae with LC_50_ = 9.30 ppm followed by *C. sinuosa* acetone extract with LC_50_ = 82.58 ppm after 72 h of exposure. At 50 and 250 ppm, *J. rubens* methylene chloride and *C. sinuosa* acetone extracts maintained 100 and 93.33% larval mortality rates, respectively. Comet assay results demonstrated the genotoxicity of both algae extracts in *C. pipiens* larvae. This study would be a beginning towards replacing chemical insecticides with algae-based bioinsecticides against *C. pipiens* mosquitos.

## Introduction

Vector-borne infectious disease transmission is an increasing problem in all countries, owing to its severe influence on human health, social life, and the economy. According to the world health organization (WHO), vector-borne infections cause over 700,000 fatalities per year, accounting for more than 17% of all infectious diseases worldwide^1^.

Mosquitoes are among the most crucial insect families that are vectors for harmful diseases and parasites^2,3^. Of these vectors, the mosquito, *Culex pipiens* Linnaeus (Culicidae) is regarded as an important insect species capable of transmitting various life-threatening human diseases^3–5^. In Egypt, it is the main transmitter of the Rift Valley fever virus, lymphatic filariasis and the West Nile virus^6–8^. Although multiple techniques have been employed to prevent mosquito-borne infections, including behavioral, chemical, and mechanical approaches, little success has been obtained due to inadequate vaccinations^9,10^.

Furthermore, the excessive use of chemical insecticides such as organophosphates, carbamates, and pyrethroids negatively impacts human health and the ecosystem and is not effective against mosquitoes’ pesticide resistance. Thus, there is a pressing demand to identify safe, environmentally acceptable, and target-specific pesticide alternatives to mosquito control^11^. Macroalgae is considered one of these alternatives^12^.

Macroalgae are regarded as one of the most significant biomass producers for various structurally distinct secondary metabolites with pharmacological and biological functions such as biofertilizer, antioxidant, antimicrobial, anticancer, and insecticides^13,14^. They contain numerous active phytochemicals including phenolics, carotenoids, flavonoids, alkaloids and terpenes that are analogous to some plants such in their potential as safe and biodegradable pesticides^15–17^.

Several studies have referred to the significant role of macroalgae extracts in pest management^18,19^. For instance, Ke-Xin et al.^20^ revealed the larvicidal activity of chloroform extracts from *Bryopsis pennata* (Bryopsidaceae) and *Sargassum binderi* (Sargassaceae) against *Aedes aegypti* larvae. Additionally, several macroalgae species are abundant along the Mediterranean coast of Alexandria (Egypt), characterized by their larvicidal action. For example, the methanolic extracts from *Codium tomentosum* (Codiaceae), *Ulva intestinales* (Ulvophyceae) and *Jania rubens* showed high larvicidal potencies against the 3rd larval instar of *C. pipiens*^21^. *Jania rubens* bioactive metabolites showed high activity as cytotoxic, antibacterial, anticancer, and molluscicide agents^22–24^. Similarly, Udayan et al.^25^ recorded the larvicidal effect of an acetone extract from *Colpomenia sinuosa* (Scytosiphonaceae) against *Artemia salina.*

Employing various extraction methods and solvents can increase the amount of bioactive chemicals in macroalgal biomass^26^. Solid-liquid extraction is the most often used process for obtaining phytochemicals from macroalgae, due to its simplicity, low cost, and time efficiency. Also, several studies referred to solvents with different polarities, such as acetone, ethanol, and methanol, to ensure maximum extraction yield^27,28^.

Detecting the effect of insecticides from algae on cell structure, DNA functions, and cell mutation in insects is crucial for determining the degree of potency of such biocontrol agents compared with that of synthetic ones^29^. In this regard, molecular biology approaches, such as comet assays, enable a novel tool to investigate ecological toxicity processes at the cellular and molecular levels. It is particularly valuable for studying insecticide resistance and to analyze terrestrial varieties of numerous categories, like insects, which are important in the human economy^30^.

Therefore, the present work originated with the aim of (i) studying the effect of different solvent extractions on the phytochemical composition of *J. rubens* and *C. sinuosa*, (ii) investigating the potential of the different extracts as larvicides against *Culex pipiens*, (iii) evaluating the molecular Genotoxicity of the promising extracts by comet assays and (iv) evaluating the correlation among different assays.

## Results

### Extraction yield 

The average yield (mg g ^− 1^ dry wt.) of each solvent extract from each macroalga is presented in (Table 1). The extract yield varied among the different solvents. For *C. sinuosa*, the maximum significant yield was observed with methanol (82 ± 0.01 mg g^− 1^), while the lowest yield was recorded with acetone (6.5 ± 0.002 mg g ^− 1^). In contrast, *J. rubens* exhibited the highest significant yield with methanol (33.9 ± 0.02 mg g ^− 1^) and the lowest with methylene chloride (0.9 ± 0.0013 mg g^− 1^).

**Table 1:** The average yield (mg g^− 1^ dry wt.) of each solvent extract from *Jania rubens* and *Colpomenia sinuosa.* Data is represented as mean ± standard error. Different letters indicate significant differences at *P* ≤ 0.05.

Macroalgae	Mean yield of extract (mg g^− 1^ dry wt.)		
	Acetone	Methanol	Methylene chloride
*Colpomenia sinuosa*	6.5 ± 0.002^b^	82 ± 0.01^a^	7.2 ± 0.0014^b^
*Jania rubens*	3.95 ± 0.001^b^	33.9 ± 0.02^a^	0.9 ± 0.0013^c^

### Phytochemical constituents

**Fig. 1:**
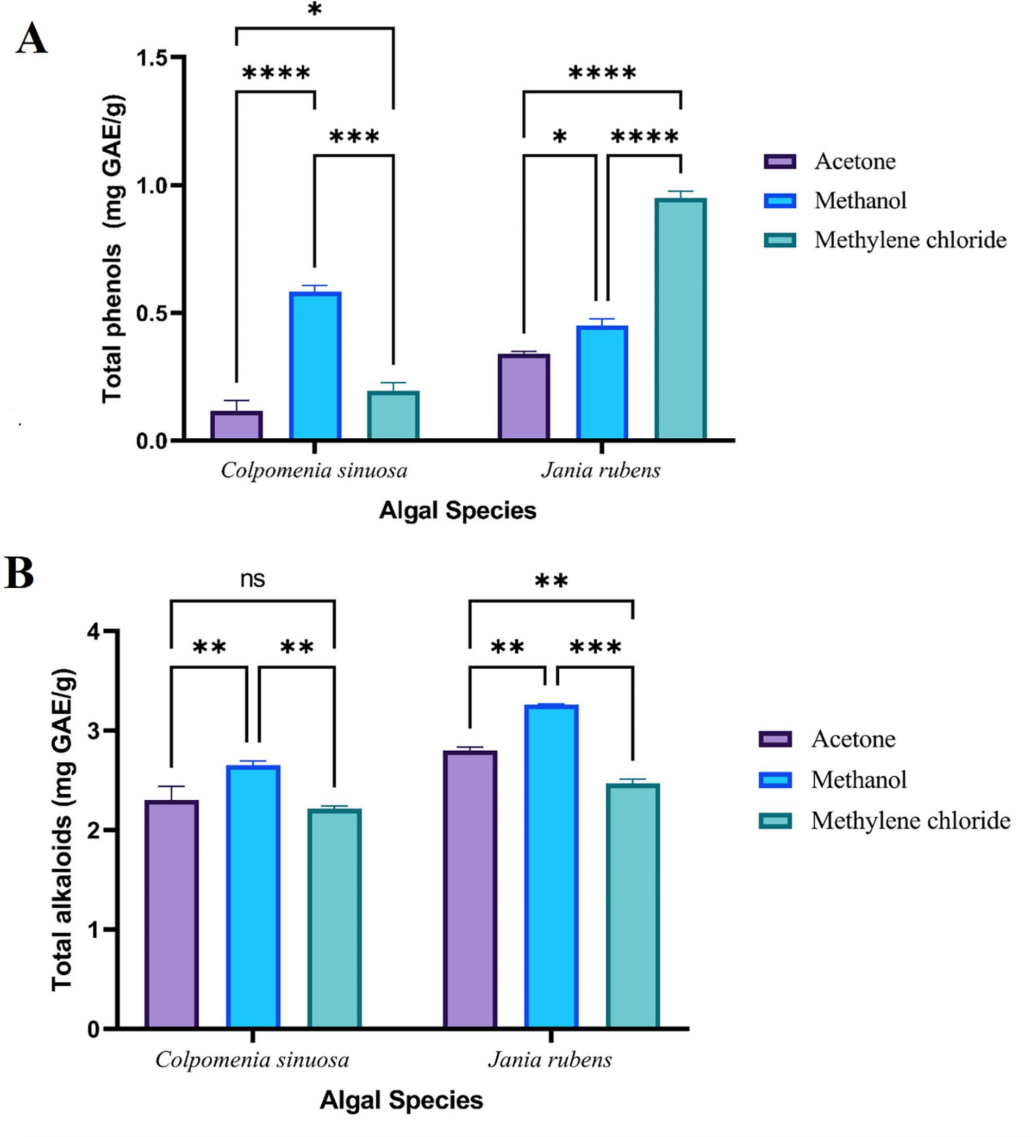
Variation in A) total phenols and B) total alkaloids contents of different solvent extracts from *C. sinuosa* and *J. rubens.* Data represents mean ± SD, *n* = 3. Two-way ANOVA according to Tukey’s multiple comparisons test. *ns* non-significant, *,**,*** and ****referred to two means significantly different at the 0.05, 0.01, 0.001 and ≤ 0.0001 levels.

**Fig. 2:**
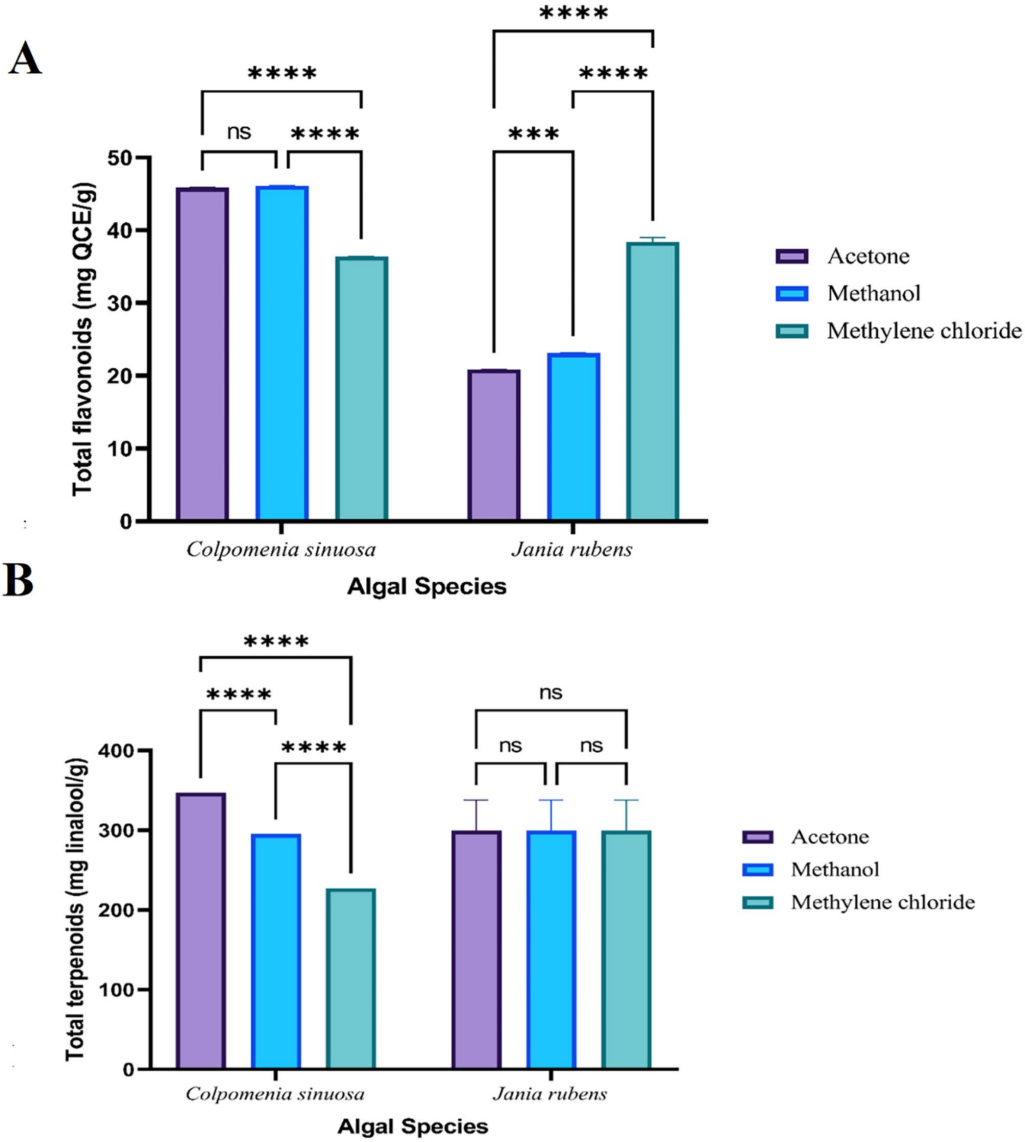
Variation in A) total flavonoids and B) total terpenoids contents of different solvent extracts from *C. sinuosa* and *J. rubens*. Data represents mean ± SD, *n* = 3. Two-way ANOVA according to Tukey’s multiple comparisons test. *ns* non-significant, *,**,*** and ****referred to two means significantly different at the 0.05, 0.01, 0.001 and ≤ 0.0001 levels.

**Fig. 3:**
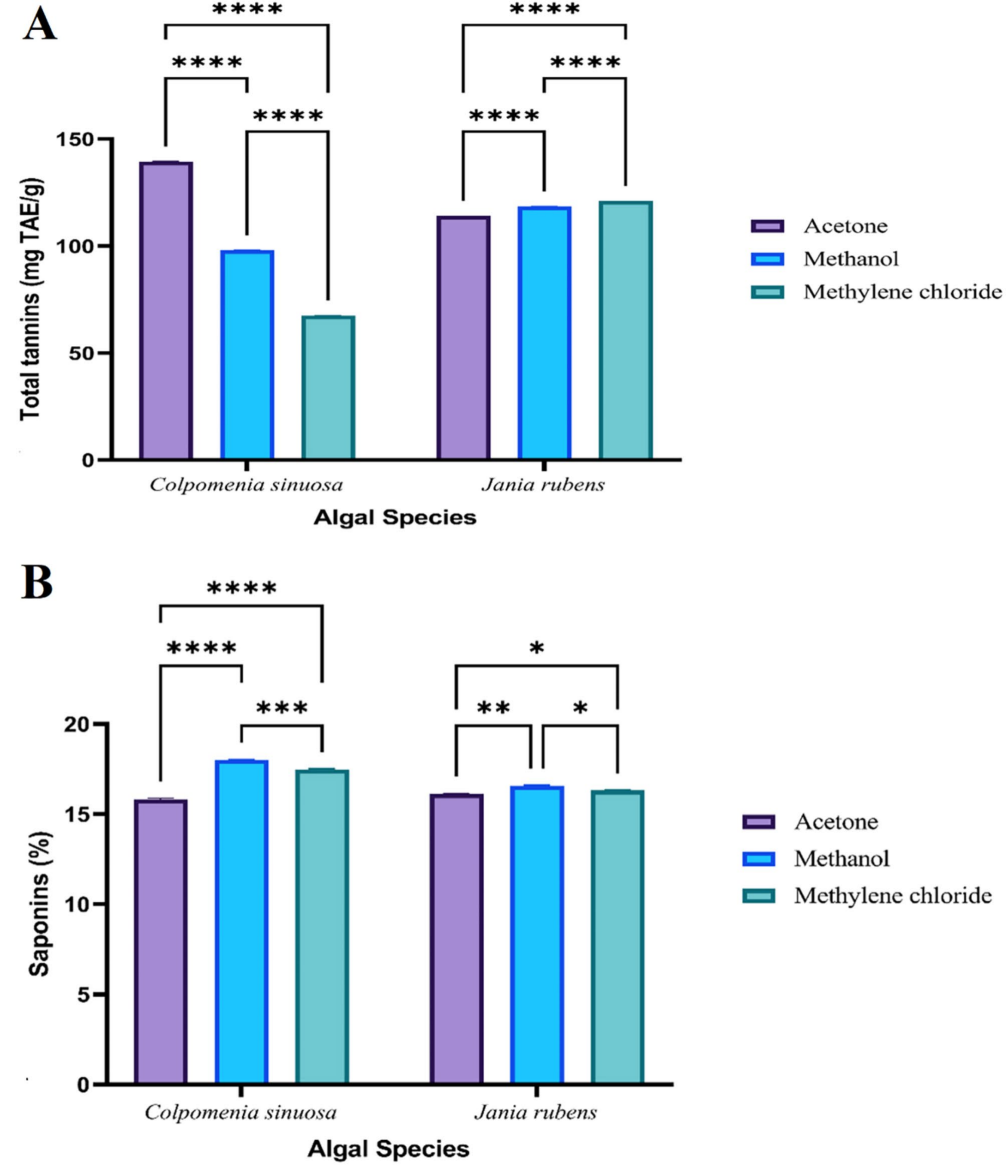
Variation in A) tannins and B) saponins content of different solvent extracts from *C. sinuosa* and *J. rubens*. Data represents mean ± SD, *n* = 3. Two-way ANOVA according to Tukey’s multiple comparisons test. *ns* non-significant, *,**,*** and ****referred to two means significantly different at the 0.05, 0.01, 0.001 and ≤ 0.0001 levels.

The variations in the total amount (mg/g extract) of phenolics, alkaloids, flavonoids, terpenoids, tannins, and saponins among the different crude extracts of *J. rubens* and *C. sinuosa* are illustrated in (Figs. 1, 2 and 3), respectively.

For *C. sinuosa*, the highest significant total phenolic content (0.583 ± 0.025 mg GAE/g) was recorded for methanol extract, and the lowest value (0.116 ± 0.04 mg GAE/g) was recorded for acetone extract. Conversely, *J. rubens* exhibited the highest significant total phenolic content (0.95 ± 0.0264 mg GAE/g) with methylene chloride extract and the lowest value (0.34 ± 0.01 mg GAE/g) was exhibited with acetone (Fig. 1A). Regarding total alkaloid content, *C. sinuosa* displayed the highest value (2.65 ± 0.04 mg GAE/g) with the methanol extract, and the lowest value (2.21 ± 0.03 mg GAE/g) with the methylene chloride extract. Conversely, *J. rubens* exhibited the highest total alkaloid content (3.26 ± 0.01 mg GAE/g) with the methanol extract, and the lowest value (2.47 ± 0.0435 mg GAE/g) with the methylene chloride extract (Fig. 1B).

Similarly, the highest significant total flavonoid content (46.13 ± 0.02 mg QCE/g) of *C. sinuosa* was recorded for methanol, and the lowermost value (36.41 ± 0.0152 mg QCE/g) was recorded for methylene chloride. While for *J. rubens*, the highest total flavonoid content (38.42 ± 0.54 mg QCE/g) was recorded for methylene chloride. The lowest value (20.88 ± 0.02 mg QCE/g) was recorded for acetone (Fig. 2A). On the contrary, the highest significant terpenoid content (346.91 ± 0.0152 mg linalool/g) for *C. sinuosa* was exhibited with acetone and the lowest value (227.11 ± 0.035 mg linalool/g) was exhibited with methylene chloride. While the highest significant terpenoid content (332.9 ± 0.04 mg linalool/g) for *J. rubens* was exhibited by methylene chloride and the lowest value (257.41 ± 0.026 mg linalool/g) was exhibited with acetone (Fig. 2B).

Meanwhile, *C. sinuosa* maintained the highest significant tannin content (139.5 ± 0.1 mg TAE/g) recorded for acetone, and the lowest (67.46 ± 0.045 mg TAE/g) was for methylene chloride. The highest significant tannins content of *J. rubens* was (121.12 ± 0.03 mg TAE/g) in the case of methylene chloride. The lowest (114.21 ± 0.02 mg TAE/g) was in the case of acetone (Fig. 3A). Saponin content recorded the highest value (18 ± 0.04%) for *C. sinuosa* with methanol and the lowest value (15.82 ± 0.06%) was recorded with acetone. Whereas *J. rubens* maintained the highest saponin content (16.58 ± 0.035%) with methanol, and the lowest value (16.12 ± 0.03%) was with acetone (Fig. 3B).

### Larvicidal activity

Tables 2 and 3, and 4 showed the larvicidal activity of *J. rubens* and *C. sinuosa* acetone, methanol, and methylene chloride extracts against the mosquito larvae of *C. pipiens* at 24, 48, and 72 h post-treatment. The data on toxicity varied depending on the species and the solvent used. For acetone extracts, the highest level of toxicity after 24 h was recorded for *J. rubens* with LC_50_ = 209.96 ppm. At the same time, *C. sinuosa* was the most toxic after 48 and 72 h, with LC_50_ values of 122.09 and 82.58 ppm, respectively (Table 2).

**Table 2:** Larvicidal activity of acetone extracts from *J. Rubens* and *C. Sinuosa* against early 3rd instar larvae of *C. pipiens*.

Macroalgae	Post-treatment	LC_25_ (*F.l. at 95%)	LC_50_ (*F.l. at 95%)	LC_90_ (*F.l. at 95%)	*Slope ± SE	*X^2^	Toxicity index	Relative potency
*C. sinuosa*	24 h	89.1 (71.28–111.38)	211.24 (168.99–264.06)	1089.23 (871.39–1361.54)	1.799 ± 0.261	17.093	39.09	1
	48 h	52.12 (41.69–65.15)	122.09 (97.68–152.62)	615.47 (492.38–769.34)	1.824 ± 0.244	21.006	67.63	1.73
	72 h	34.44 (27.55–43.05)	82.58 (66.06–103.22)	434.92 (347.94-543.65)	1.776 ± 0.242	21.452	100	2.56
*J. rubens*	24 h	136.84 (94.71–165.17)	209.96 (174.97–307.72)	473.57 (378.859–591.9675)	3.628 ± 0.391	7.8514	39.33	1.01
	48 h	90.46 (72.37–113.08)	153.76 (123.01–192.2)	421.28 (337.03–526.61)	2.928 ± 0.288	17.372	53.71	1.37
	72 h	61.15 (48.92–76.43)	108.65 (86.92–135.81)	323.90 (259.12–404.88)	2.702 ± 0.262	27.228	76.01	1.94

*(F.l.) Fiducially Limits.

*(X^2^) Chi-square value.

*Slope of the concentration-inhibition regression line ± standard error, Values are significantly different at *p* < 0.05.

**Table 3:** Larvicidal activity of methanol extracts from *J. Rubens* and *C. Sinuosa* against early 3rd instar larvae of *C. pipiens*.

Macroalgae	Post-treatment (h)	LC_25_ (*F.l. at 95%)	LC_50_ (*F.l. at 95%)	LC_90_ (*F.l. at 95%)	*Slope ± SE	*X^2^	Toxicity index	Relative potency
*C. sinuosa*	24	660.79 (528.64–825.99)	2456.28 (1965.03–3070.35)	29765.91 (23812.72–37207.38)	1.183 ± 0.407	1.359	15.53	1.58
	48	332.82 (226.98–416.0312)	1388.71 (1110.97–1735.88)	20960.78 (16768.62–26200.97)	1.087 ± 0.318	0.7999	27.48	2.79
	72	113.27 (79.66–144.25)	446.81 (357.45–558.51)	6061.83 (4849.46–7577.28)	1.132 ± 0.2602	2.4523	85.39	8.68
*J. rubens*	24	793.53 (634.83–991.91)	3876.94 (3101.55–4846.18)	78976.51 (63181.21–98720.63)	0.979 ± 0.378	0.593	9.8	1
	48	139.34 (90.77–215.24)	921.08 (736.86–1151.35)	33327.19 (26661.75–41658.99)	0.822 ± 0.261	1.9953	41.42	4.21
	72	36.68 (29.35–45.86)	381.55 (305.24–476.94)	32656.12 (26124.89–40820.15)	0.663 ± 0.236	1.879	100	10.16

*(F.l.) Fiducially Limits.

*(X^2^) Chi-square value.

*Slope of the concentration-inhibition regression line ± standard error, Values are significantly different at *p* < 0.05.

**Table 4:** Larvicidal activity of methylene chloride extracts from *J. Rubens* and *C. Sinuosa* against early 3rd instar larvae of *C. pipiens*.

Macroalgae	Post-treatment (h)		LC_25_ (*F.l. at 95%)	LC_50_ (*F.l. at 95%)	LC_90_ (*F.l. at 95%)	*Slope ± SE	*X^2^	Toxicity index	Relative potency
*C. sinuosa*	24		660.79 (528.64–825.99)	2456.28 (1965.03–3070.35)	29765.91 (23812.72–37207.38)	1.183 ± 0.407	1.3597	0.38	1.00
	48		250.72 (206.87–352.06)	553.87 (383.31–1221.28)	2497.09 (1997.67–3121.36)	1.959 ± 0.385	3.693	1.68	4.43
	72		123.14 (96.07–149.93)	370.42 (271.94–688.02)	3002.48 (2401.98–3753.09)	1.410 ± 0.270	3.957	2.51	6.63
*J. rubens*	24		11.52 (9.22–14.40)	21.178 (16.94–26.47)	67.32 (53.86–84.15)	2.552 ± 0.258	24.517	43.93	115.98
	48		7.53 (6.03–9.42)	14.093 (11.27–17.62)	46.33 (37.07–57.92)	2.479 ± 0.261	19.869	66.02	174.29
	72		4.51 (2.61–6.34)	9.30 (6.72–11.51)	36.76 (30.91–47.09)	2.148 ± 0.276	7.556	100	264.01

*(F.l.) Fiducially Limits.

*(X^2^) Chi-square value.

*Slope of the concentration-inhibition regression line ± standard error, Values are significantly different at *p* < 0.05.

.

In the case of methanol extracts, *C. sinuosa* was the most effective one with LC_50_ = 2456.28 ppm after 24 h, whereas after 48 and 72 h, *J. rubens* was the most effective one with LC_50_ = 921.09 and 381.55 ppm, respectively (Table 3). However, in the case of methylene chloride extracts, *J. rubens* was the most toxic species with LC_50_ of 21.18, 14.09, and 9.30 ppm after 24, 48, and 72 h, respectively, and *C. sinuosa* was the lowest one with LC_50_ of 2456.283, 553.877, and 370.420 ppm after 24, 48, and 72 h, respectively, as shown in (Table 4).

Also, as listed in Table 5, the mortality percentage was the highest (96.67%) after 24 h in the case of methylene chloride extract from *J. rubens* at 50 ppm. After 48 h, the mortality percentage was 100% at 50 ppm, using the methylene chloride extract from *J. rubens*. At the end of the post-treatment (72 h), the acetone extract from *J. rubens* showed a 96.67% mortality at 250 ppm and 93.33% for *C. sinuosa* at 250 ppm.

**Table 5:** Mortality percentage of *J. Rubens* and *C. Sinuosa* extracts against early 3rd instar larvae of *Culex pipiens* at different concentrations. Data are represented as mean ± standard deviation.

Treatments	% Mortality											
	Acetone				Methanol				Methylene chloride			
	Conc. (ppm)^a^	24 h	48 h	72 h	Conc. (ppm)	24 h	48 h	72 h	Conc. (ppm)	24 h	48 h	72 h
*Colpomenia sinuosa*	250	70 ± 10	86.67 ± 15.28	93.33 ± 11.5	250	13.3 ± 5.77	23.3 ± 5.77	43.33 ± 5.77	250	13.3 ± 5.77	30 ± 0	46.67 ± 5.77
	200	36.67 ± 5.77	56.67 ± 11.54	70 ± 10	200	10 ± 0	16.67 ± 5.77	33.33 ± 5.77	200	10 ± 0	16.67 ± 5.77	33.3 ± 11.5
	150	33.3 ± 11.55	46.67 ± 5.77	60 ± 10	150	6.67 ± 5.77	13.3 ± 5.77	26.67 ± 5.77	150	6.67 ± 5.77	10 ± 10	23.33 ± 5.77
	100	26.67 ± 5.77	40 ± 10	46.67 ± 5.77	100	3.3 ± 5.77	10 ± 10	20 ± 10	100	3.3 ± 5.77	6.67 ± 5.77	20 ± 10
	50	16.67 ± 5.77	30 ± 0	43.33 ± 5.77	50	3.3 ± 5.77	6.67 ± 5.77	16.67 ± 5.77	50	3.3 ± 5.77	3.3 ± 5.7	13.3 ± 5.77
*Jania rubens*	250	66.67 ± 5.77	83.3 ± 5.77	96.67 ± 5.77	250	13.3 ± 5.77	36.67 ± 5.77	50 ± 10	50	96.67 ± 5.77	100 ± 0	100 ± 0
	200	46.67 ± 5.77	63.3 ± 5.77	73.33 ± 5.77	200	10 ± 0	26.67 ± 5.77	40 ± 0	40	70 ± 17.3	86.67 ± 15.28	96.67 ± 5.77
	150	23.3 ± 5.77	36.67 ± 5.77	53.33 ± 5.77	150	6.67 ± 5.77	23.33 ± 5.77	36.67 ± 5.77	30	53.33 ± 5.77	70 ± 10	83.3 ± 5.77
	100	10 ± 10	23.3 ± 5.77	36.67 ± 5.77	100	6.67 ± 5.77	20 ± 10	33.33 ± 11.3	20	43.3 ± 5.77	56.67 ± 5.77	70 ± 0
	50	3.3 ± 5.77	13.3 ± 5.77	26.67 ± 5.77	50	3.3 ± 5.77	16.67 ± 5.77	30 ± 10	10	26.67 ± 5.77	43.3 ± 5.77	56.67 ± 5.77
DMSO (Control)	–	0.0 ± 0.0	0.0 ± 0.0	0.0 ± 0.0	–	0.0 ± 0.0	0.0 ± 0.0	0.0 ± 0.0	–	0.0 ± 0.0	0.0 ± 0.0	0.0 ± 0.0
Distilled water (Blank)	–	0.0 ± 0.0	0.0 ± 0.0	0.0 ± 0.0	–	0.0 ± 0.0	0.0 ± 0.0	0.0 ± 0.0	–	0.0 ± 0.0	0.0 ± 0.0	0.0 ± 0.0

^a^ppm = part per million.

### GC/MS analysis

**Fig. 4:**
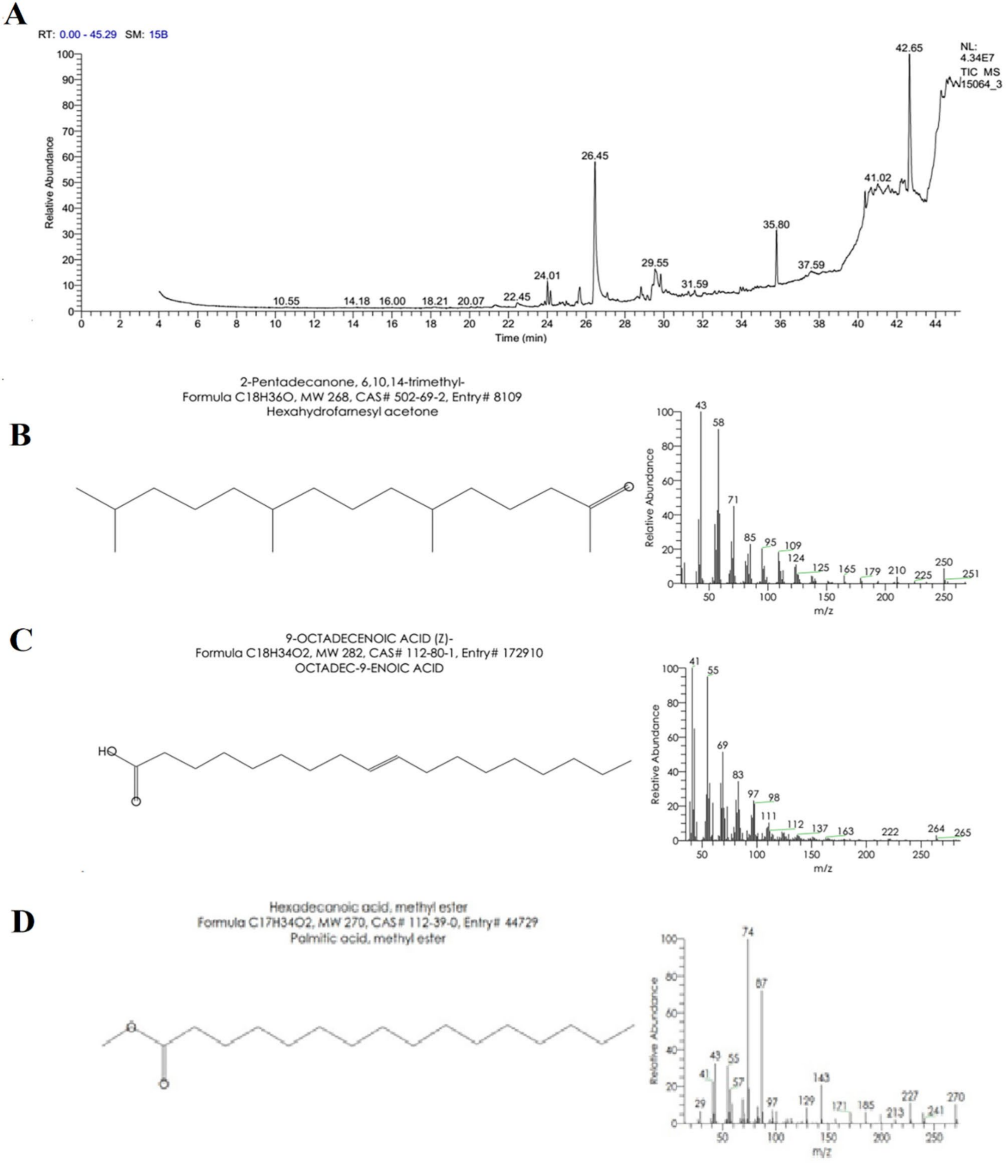
(**A**) GC-MS chromatogram for methylene chloride extract of *J. rubens*. Structure, formula, molecular weight, CAS number and the relative abundance of the three abundant metabolites peaks were identified as (**B**) 2-pentadecanone, 6,10,14-trimethyl- (**C**), 9-octadecenoic acid (Z)- (**D**) hexadecanoic acid, methyl ester.

*J. rubens* methylene chloride extract GC/MS results revealed 37 peaks corresponding to 37 phytochemical compounds, although only 16 were identified (Table 6). Hexadecanoic acid, methyl ester (44.52%), 9-octadecenoic acid (Z)- (9.19%), and 2-pentadecanone, 6,10,14-trimethyl- (6.19%) were the most prevalent chemicals (Fig. 4).

**Table 6:** GC/MS analysis of *J. rubens* methylene chloride extract.

No.	Peak no.	Compound	Retention time (min.)	Chemical group	Formula	Molecular weight	Area %
1	1	2-Pentadecanone, 6,10,14-trimethyl-	24.01	Sesquiterpenoids	C_18_H_36_O	268	6.19
2	2	Neophytadiene	24.16	Sesquiterpene lactone	C_20_H_38_	278	3.73
3	3	Z-(13,14-Epoxy)tetradec-11-en-1-ol acetate	24.01	Diverse functional group	C_16_H_28_O_3_	268	1.23
4	4	Hexadecanoic acid, methyl ester	26.45	Fatty acid	C_17_H_34_O	270	44.52
5	5	Hexadecanoic acid, 2,3-dihydroxypropyl ester	27.09	Fatty acid methyl ester	C_19_H_38_O_4_	330	4.28
6	6	9- octadecenoic acid (Z)-	28.82	Fatty acid	C_18_H_34_O_2_	282	9.19
7	7	E-10,13,13-Trimethyl-11-tetradecen-1-ol acetate	29.14	Ester	C_19_H_36_O_2_	296	1.88
8	8	Cyclopropanebutanoic acid, 2-[[2-[[2-[(2-pentylcyclopropyl)meth yl]cyclopropyl]methyl]cyclopropyl] methyl]-, methyl ester	29.39	Methyl ester	C_25_H_42_O_2_	374	2.52
9	10	Geranyl oleate	29.84	Ester	C_28_H_50_O_2_	418	4.14
10	11	Ethyl iso-allocholate	31.26	Steroid	C_26_H_44_O_5_	436	1.73
11	12	Isochapin B	31.59	Sesquiterpene lactone	C_19_H_22_O_6_	336	1.97
12	13	Linoleic acid ethyl ester	33.93	Ester	C_20_H_36_O_2_	308	1.95
13	14	1-Heptatriacotanol	34.07	Alcohol	C_37_H_76_O	537	1.47
14	16	Cholesta-4,6-dien-3-ol, (3á)-	40.36	Sterol	C_27_H_44_O	384	5.42
15	17	7,8-Epoxylanostan-11-ol,3-acetoxy-	40.55	Alcoholic compound	C_32_H_54_O_4_	502	3.23
16	21	Oleic acid, 3-(octadecyloxy)propyl	41.02	Diverse functional group	C_39_H_76_O_3_	593	5.14

**Fig. 5:**
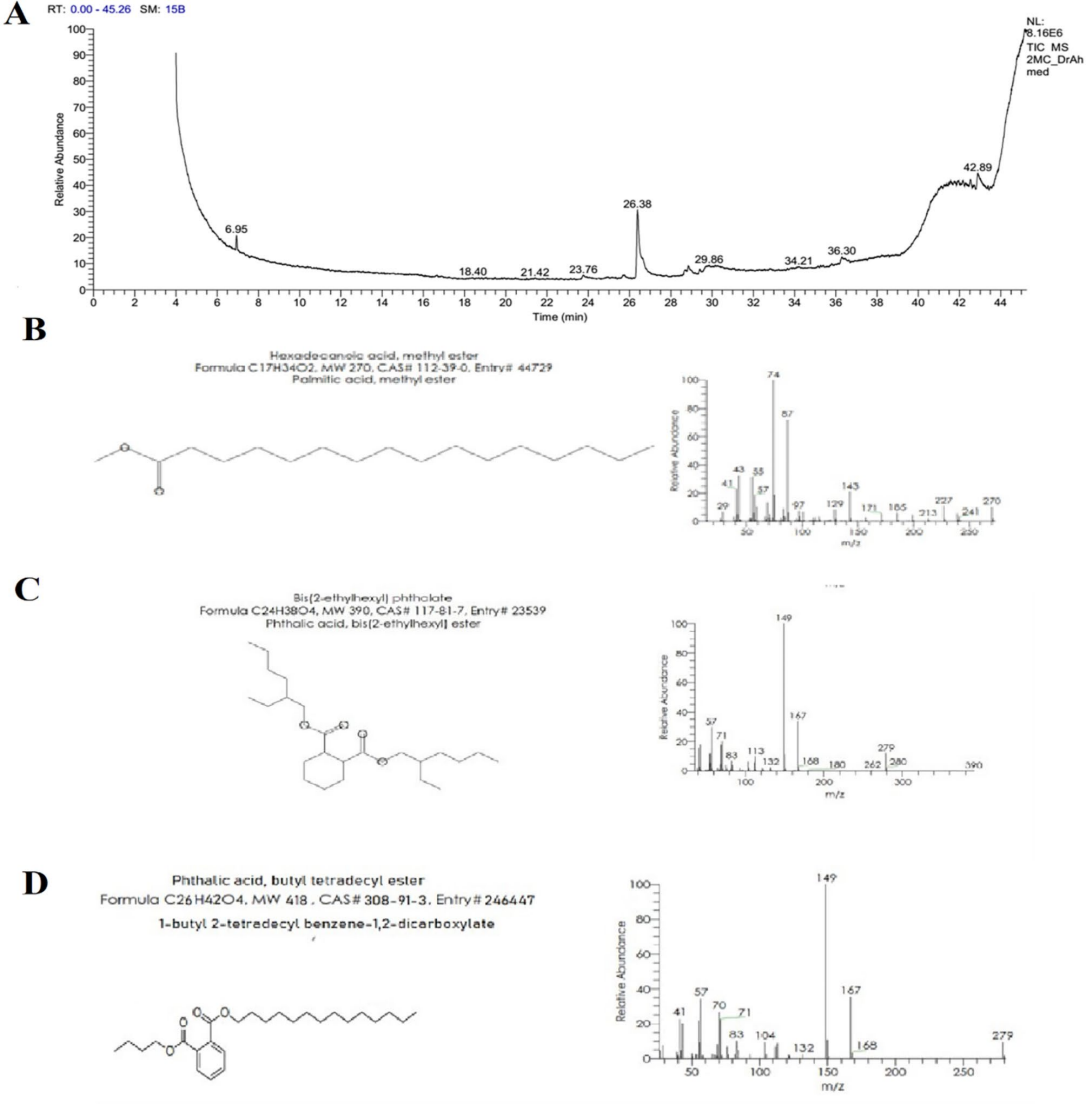
(**A**) GC-MS chromatogram for acetone extract of *C. sinuosa.* Structure, formula, molecular weight, CAS number and the relative abundance of the three abundant metabolites peaks were identified as (**B**) hexadecanoic acid, methyl ester; (**C**) Bis(2-ethylhexyl) phthalate and (**D**) phthalic acid, butyl tetradecyl ester.

While the GC/MS results of *C. sinuosa* acetone extracts revealed 18 peaks corresponding to 18 phytochemical compounds, only 7 were identified and characterized (Table 7). The 3 major highest-peak chemical compounds were hexadecanoic acid, methyl ester (41.3%), phthalic acid, butyl tetradecyl ester (19.87%), and Bis (2-ethylhexyl) phthalate (10.09%) (Fig. 5).

**Table 7:** GC/MS analysis of *C. Sinuosa* acetone extract.

No.	Peak no.	Compound	Retention time (min.)	Chemical group	Formula	Molecular weight	Area %
1	3	2-Pentadecanone, 6,10,14-trimethyl-	24.77	Sesquiterpene	C_18_H_36_O	268	5.78
2	4	Hexadecanoic acid, methyl ester	26.83	Fatty acid ester	C_17_H_34_O	270	41.3
3	5	Phthalic acid, butyl tetradecyl ester	28.84	Phenolic acid	C_26_H_42_O_4_	418	19.09
4	6	Bis(2-ethylhexyl) phthalate	35.00	Fatty acid ester	C_24_H_38_O_4_	390	10.09
5	11	Indole-2-one, 2,3-dihydro-N-hydroxy-4-methoxy-3,3-dimethyl-	37.243	Indole alkaloid	C_11_H_13_NO_3_	207	6.7
6	12	1,25-Dihydroxy vitamin D3	42.14	Vitamin	C_27_H_44_O_3_	416	7.09
7	13	1-(2-Acetoxyethyl)-3,6-diazahomoad amantan-9-one oxime	42.53	alkaloid	C_13_H_21_N_3_O_3_	267	9.18

### Molecular genotoxicity assessment

**Fig. 6:**
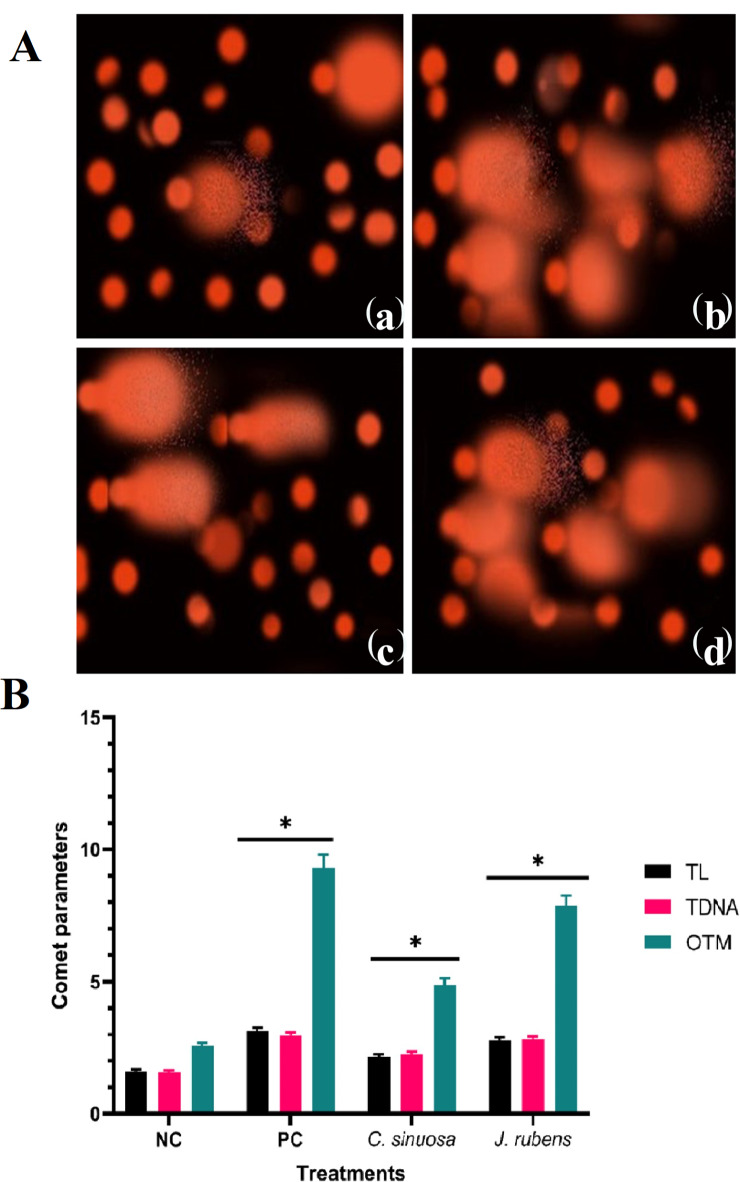
(**A**) Comparison of comet assay profile for genotoxicity in late 3rd larval instar of *C. pipiens*; (**a**) NC (DMSO), (**b**) PC (Malathion 5), (**c**) *C. sinuosa* (acetone extract), (**d**) *J. rubens* (methylene chloride extract). (**B**) Comet assay parameters (tail length (TL), tail DNA (T DNA %) and tail moments (OTM) under different treatments. Error bars represented the standard deviation, data were calculated by *t*- test analysis, *referred to two means significantly different at the 0.05 level.

DNA damage in *C. pipiens* cells treated with *C. sinuosa* and *J. rubens* extracts was quantified using the comet assay and demonstrated in tail length (TL), tail DNA% (T DNA), and tail moment (OTM) (Fig. 6). The degree of damage to DNA varied between treated and controlled *C. pipiens* larvae. The negative control DMSO-treated body cells had virtually rounded nuclei (Fig. 6A(a)). Nuclei with a visible tail-like extension were found in the body cells of mosquitoes treated with positive control (Malathion 5) (Fig. 6A(b)), indicating that the insect’s body cells had been damaged and DNA strand breakage had occurred. Malathion 5 treatment was observed to generate a considerable increase in the levels of TL in *C. pipiens* body cells. The third larval instar showed a significant increase in DNA TL (3.14 μm) in response to insecticide compared to the negative control (1.61 μm) (Fig. 6B). DNA damage to body cells was demonstrated by T DNA% values of *C. pipiens* (2.97%), significantly greater than the control flies (1.59%) (Fig. 6B). It was also discovered that the insecticide treatment increased the levels of OTM (9.33) in *C. pipiens* body cells much more than the control levels (2.56) (Fig. 6B). Furthermore, DNA damage was found in *C. pipiens* body cells treated with *C. sinuosa* acetone extract and the methylene chloride extract of *J. rubens* (Fig. 6A(c)) and (Fig. 6A(d)). For *C. sinuosa* and *J. rubens* extracts, the TL values were (2.16 and 2.79 μm), the T DNA% was (2.26 and 2.82%), and the OTM values were (4.88 and 7.87), respectively (Fig. 6B).

### Intercorrelation estimation

#### The effect of both *C. Sinuosa* and *J. Rubens* extracts via different parameters: cell plot

**Fig. 7:**
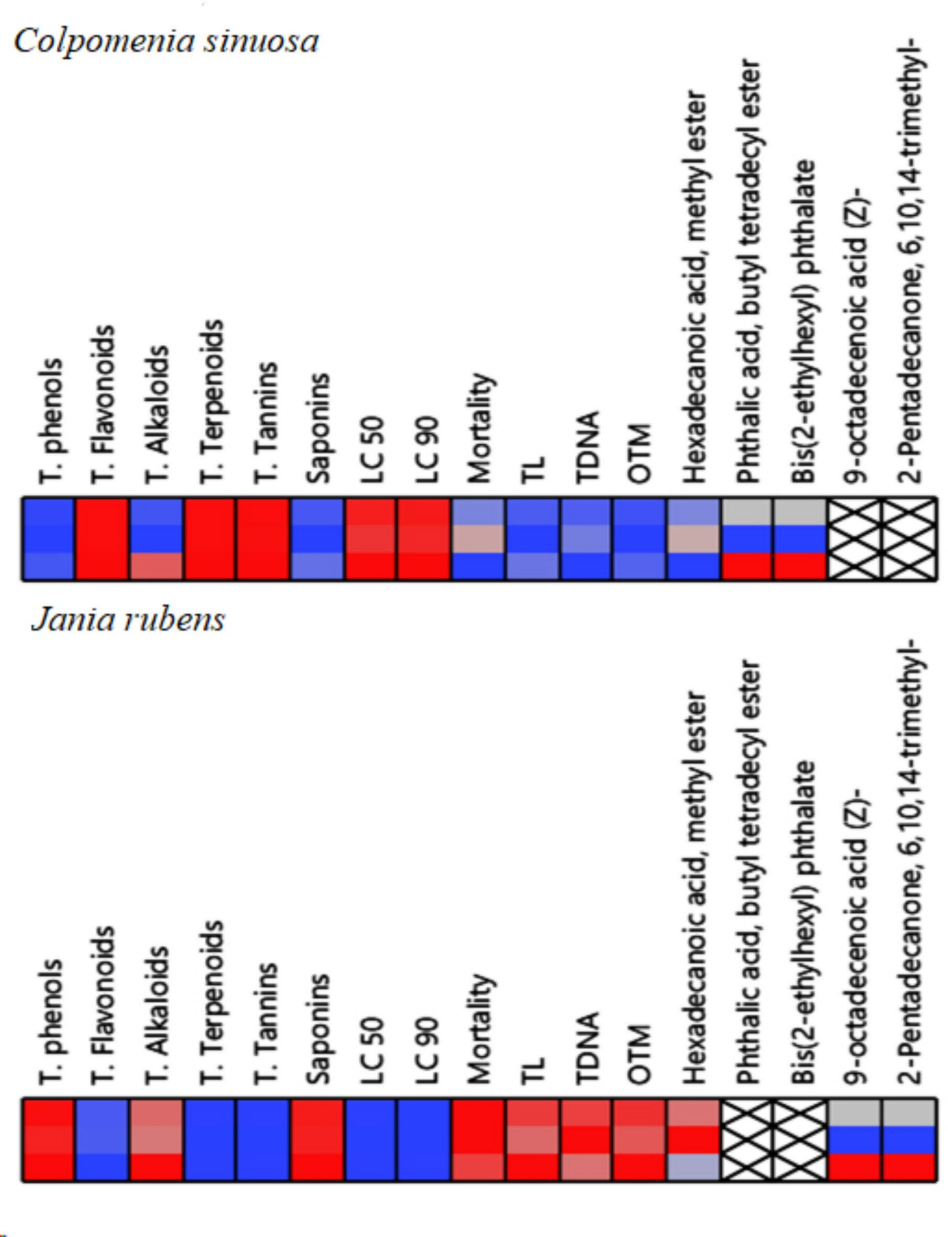
Cell plot of 17 traits of phytochemical and some selected metabolites of GC/MS analysis of *C. sinuosa* (acetone extract), and *J. rubens* (methylene chloride extract) and their larvicidal activity (LC_50_ and LC_90_; larvicidal potency and mortality percentage), comet parameters (TL tail length, TDNA tail DNA, OTM tail moment) on late 3rd larval instar of *C. pipiens.* The red color assumes the highest value, while the blue color assumes the lowest one.

As shown in (Fig. 7), the cell plot of 17 studied parameters was assessed in which the red color assumed the highest value, while the blue color assumed the lowest one. For the phytochemical analysis, the acetone extract of *C. sinuosa* showed the highest T. flavonoids, T. terpenoids, and T. tannins. In contrast, the methylene chloride extract of *J. rubens* had the maximum T. phenols, alkaloids, and T. saponins. According to GC/MS profiling results, some valuable metabolites varied in a different pattern in both algal extracts. Hexadecanoic acid, methyl ester showed the maximum content in *J. rubens* extract, and 2-pentadecanone, 6,10,14-trimethyl- and 9-octadecenoic acid (Z)- were shown in this extract only.

Meanwhile, phthalic acid, butyl tetradecyl ester and Bis (2- ethyl hexyl) phthalate were found only in *C. sinuosa* extract. According to larvicidal potency (LC_50_ and LC_90_), *C. sinuosa* extract showed the highest activity compared to *J. rubens* extract, however, the latter showed the maximum mortality percentage. For the genotoxicity assessment, *J. rubens* extract recorded the highest values of the three comet parameters (TL, T DNA% and OTM).

#### Multivariate analysis

##### Scatter plot matrix with correlation circles

**Fig. 8:**
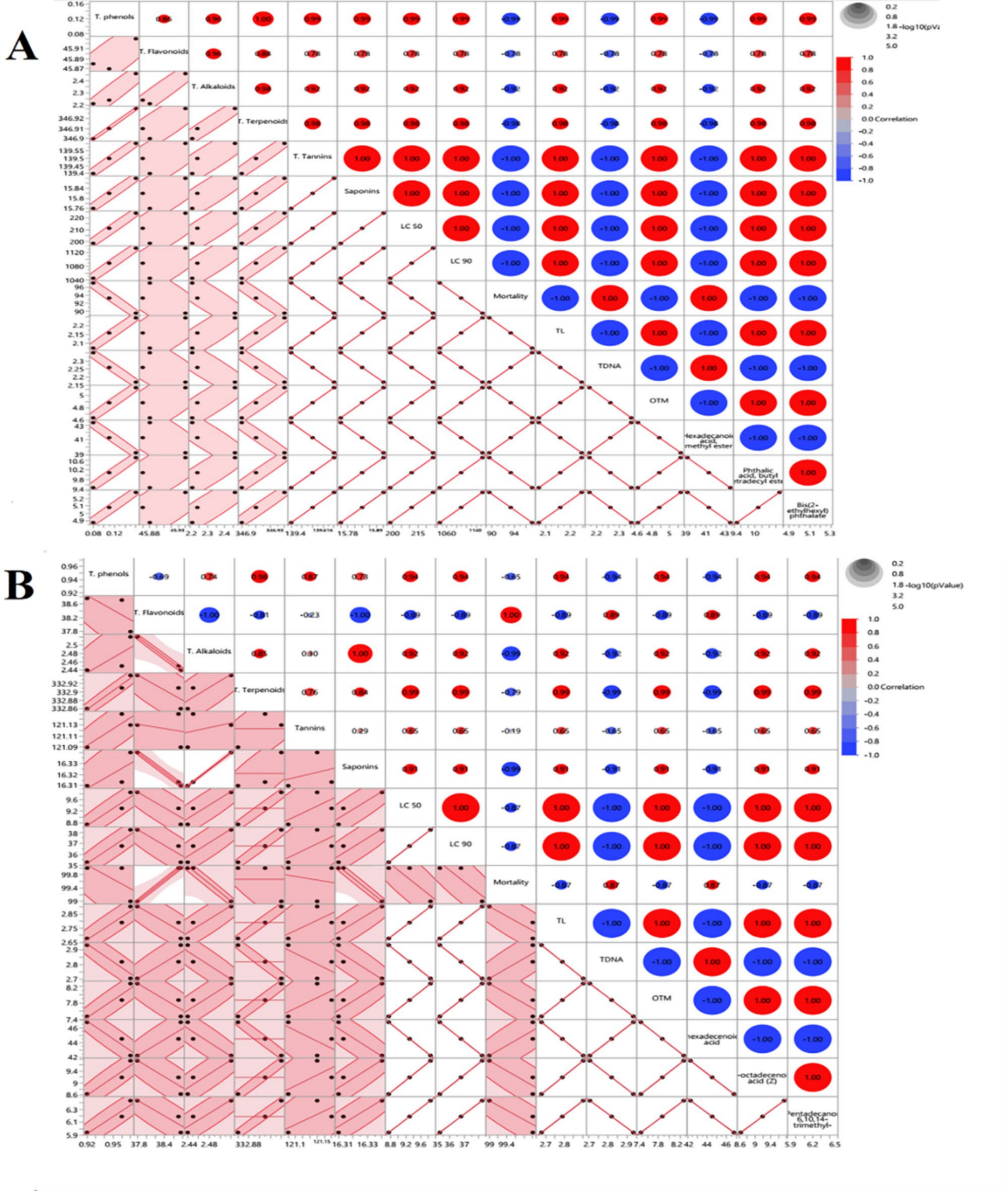
Scatter plot and heatmap correlation circles among different phytochemical parameters and some selected metabolites of GC/MS analysis of (**A**) *C. sinuosa* (acetone extract), and (**B**) *J. rubens* (methylene chloride extract) and their larvicidal and Genotoxicity activities on late 3rd larval instar of *C. pipiens*. Red color indicates positive correlation, blue color indicates the negative correlation, while the size of circles indicates the significance (see scale at the above right corner).* LC*_*50*_* and LC*_*90*_ larvicidal potency, TL tail length, T DNA tail DNA%, OTM tail moment.

Scatter plot matrix with heatmap correlation circles of 17 quantitative parameters (GC/MS metabolic measurements of the acetone extract of *C. sinuosa* and the methylene chloride extract of *J. rubens* and their phytochemical composition and their larvicidal and genotoxicity activities on the late 3rd larval instar of *C. pipiens.* The scatter plot matrix displayed the density ellipses in each scatter plot, with the red circles representing about 95% of the data, as shown in (Fig. 8). The red color denoted a positive correlation, the blue color represented a negative correlation, and the size of the circles was proportionate to the correlation strength. In *C. sinuosa* extract, T. phenols were positively correlated with T. flavonoids and negatively correlated to the rest of the phytochemical contents, as represented in (Fig. 8A). Highly strong positive correlations were detected among T. phenols, T. flavonoids, T. alkaloids, T. terpenoids, T. tannins, and T. saponins contents. In the same trend in larvicidal activity, LC_50_ and LC_90_ values were positively correlated to all phytochemical contents and were negatively correlated with mortality percentage.

Among the three comet parameters, TL was strongly positively correlated to OTM and was negatively correlated with T DNA%. Similarly, TL and OTM were strongly positively correlated to LC_50_ and LC_90_ and all other phytochemical compounds. For GC/MS metabolites, phthalic acid content was strongly correlated to butyl tetradecyl ester and T. phenols. In the same trend, Bis (2- ethyl hexyl) phthalate was strongly positively correlated to phthalic acid, T. saponins, T. tannins, LC_50_, LC_90_, TL, OTM and all the other phytochemical compounds. Hexadecanoic acid methyl ester content was strongly positively correlated with the mortality and T DNA percentages, as observed in (Fig. 8A).

In *J. rubens* extract (Fig. 8B), T. phenols were positively correlated to T. terpenoids, T. tannins, and T. saponins and were negatively correlated with T. flavonoids. Also, T. phenols were positively correlated to LC_50_, LC_90_, TL, OTM, 2-pentadecanone, and 9-octadecenoic acid (Z)- and were negatively correlated to mortality %, T DNA%, and hexadecanoic acid methyl ester. Similarly, T. flavonoids were negatively correlated to all phytochemical compounds and strongly correlated to mortality %, T DNA%, and hexadecanoic acid methyl ester. T. tannins and T. saponins were positively correlated with other parameters such as (LC_50_, LC_90_, TL, OTM, 9-octadecenoic acid (Z)- and 2-pentadecanone. There were strong correlations among LC_50_, LC_90,_ and TL, OTM values for the larvicidal and genotoxicity activities. For GC-MS metabolites, hexadecanoic acid methyl ester content was strongly positively correlated to T. flavonoids, T DNA% and mortality%. At the same time, 2-pentadecanone was positively correlated to 9-octadecenoic acid (Z) and was negatively correlated to hexadecanoic acid methyl ester. While 2-pentadecanone and 9-octadecenoic acid (Z)- had a strong positive correlation with TL, OTM, LC_50_, LC_90,_ and all the phytochemical compounds except T. flavonoids.

##### Principal component analysis (PCA) and biplot

**Fig. 9:**
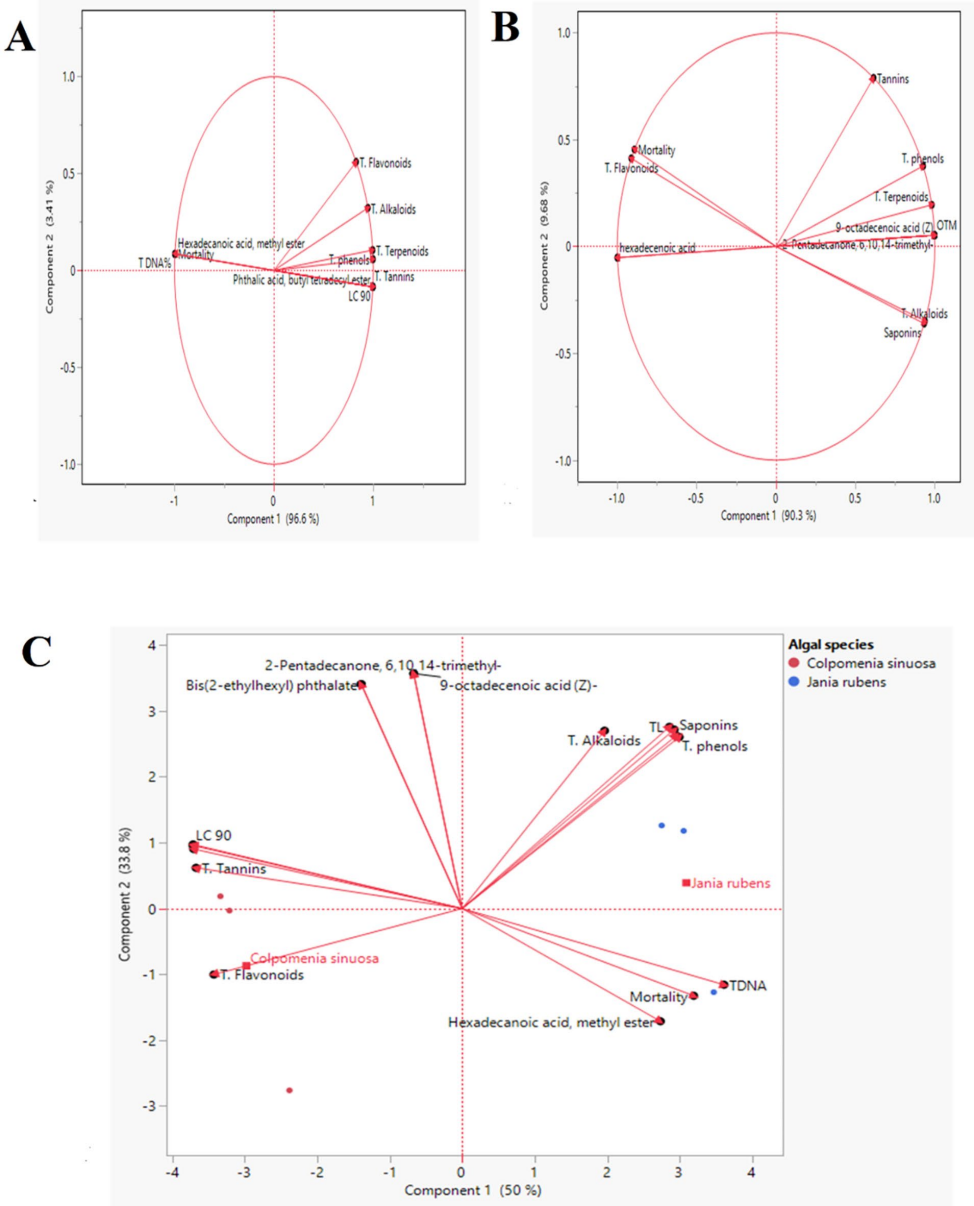
Principal component analysis (PCA); (**A**,**B**) scatter plot of *C. sinuosa* (acetone extract), and *J. rubens* (methylene chloride extract), respectively; and (**C**) biplot based on PC1 and PC2 components illustrating the effect of all combined data (phytochemical, selected metabolites of GC/MS analysis, larvicidal and genotoxicity activities of both algal extracts on late 3rd larval instar of *C. pipiens*. The red dots were algal extracts, and the vectors (red arrows) were parameters. The abbreviations were previously mentioned in the earlier figures.

The length and the cosine of the vector’s angle were employed in the system of the two first components to discriminate between each algal extract individually. Both algal extracts in the biplot (Fig. 9). The PCA scatter plot of *C. sinuosa* extract, the first component PC1 accounted for 96.6% of the total variation. In contrast, the second component PC2 accounted for 3.41% (Fig. 9A). The acute angle between the following vectors: T. flavonoids and T. alkaloids, T. phenols and T. terpenoids, TL & OTM and LC_50_ & LC_90_ with phthalic acid which establishes a significant strong positive relationship between these attributes and was found as the most effective parameters in differentiation between vectors of *C. sinuosa* extract. On the contrary, there was a negative relationship between LC_50_ and LC_90_ with mortality %, and hexadecanoic acid methyl ester methyl ester was negatively correlated to all phytochemical compounds.

In the *J. rubens* extract PCA scatter plot, the first component PC1 scored 90.3%. The second component PC2 scored 9.68% of the total variation observed (Fig. 9B). The vectors of T. phenols and T. terpenoids formed an acute angle with each other as well as with 2-pentadecanone and 9-octadecenoic acid (Z), indicating a strong positive association. Obtuse angles were found between T. tannins and T. phenols, proving a weak correlation.

Figure 9C showed the biplot, which differentiated between *C. sinuosa* and *J. rubens* extracts. The first component, PC1, was recorded as 50% and the second component, PC2, as 33.8% of the total variation. The most effective vectors in *C. sinuosa* extract were T. flavonoids, T. terpenoids, T. tannins, LC_90_, and hexadecanoic acid methyl ester. On the contrary, 2-pentadecanone, 6,10,14-trimethyl-, 9-octadecenoic acid (Z)-, TL, OTM, T. saponins, T. phenols, T. alkaloids and hexadecanoic acid methyl ester were found to be the most influential parameters in differentiation between *J. rubens* extract. The PCA multivariate analysis confirmed the heatmap correlation data that had been reported before.

## Discussion

Macroalgae are recognized as a prospective source of bioactive secondary metabolites such as tannins, alkaloids, phenols, saponins, terpenoids, and flavonoids that have great potentials in many areas, including agriculture, medicine, cosmetics, and biocontrol agents^31,32^. Because of the growing demand for bioactive natural chemicals and the variation in their quantities among different macroalgae species, selecting the proper solvent and extraction process is critical for assuring effective extraction^33^. Accordingly, the present study originated to enhance bioactive metabolite extraction from *Jania rubens* and *Colpomenia sinuosa* through different solvents for potential use as genotoxic and larvicides for mosquito larvae.

The present results demonstrated variations in the extracts yields of both *C. sinuosa* and *J. rubens* which may be attributed to differences in their growth habitat, the nature and amount of biocompounds present in each species^34^. Also, the high extraction yield of both *C. sinuosa* and *J. rubens* exhibited with methanol is in harmony with the investigations of^35^, which found that *Padina tetrastromatica* (Dictyotaceae) and *Gracilaria tenuistipitata* (Florideophyceae) showed maximum metabolite yield with methanol compared to ethanol and water. Moreover, methanol is an efficient solvent for polyphenol and antioxidant extraction from various sources including plants presumably because it has a greater dielectric constant than ethanol, acetone, and chloroform^36,37^. Furthermore, saponins are polar chemicals easily extracted using high-polarity solvents such as methanol.

The experimental results proved the maximum contents of total alkaloids, phenolics, tannins, and terpenoids in the case of *J. rubens* methylene chloride extract. It has been documented by Akinmoladun et al.^38^ that plant extract yield and antioxidant activity are affected by the solvent used in extraction. In this regard, methylene chloride solvent is classified as a mid-polar solvent used to extract polyphenols and other mid-polar phytochemicals. Our results are in harmony with those obtained by^39^, who examined the effect of methylene chloride on phytochemical screening and antioxidant activity of *Strychnos henningsii* (Loganiaceae) and *Ficus sycomorus* (Moraceae) and documented its effectiveness in saponins, flavonoids, alkaloids, steroids and terpenoids extraction with high amounts.

The high contents of terpenoids and tannins observed in the acetone extraction of *C. sinuosa* may be attributed to the excellent solubility of these compounds in acetone. Terpenoids are known for being non-polar compounds that typically require non-polar or partially polar solvents for effective extraction^40^.

The promising results of the larvicidal activity assay underscored the potential efficacy of *C. sinuosa* and *J. rubens* extracts as larvicides for controlling *C. pipiens* larvae. The differences in larvicidal activity among the different macroalgae extracts might be attributed to levels of active constituents in each extract. In this regard algal extracts have varying larvicidal properties according to the species of the target insect and their developmental stage. Previous findings are partially consistent with other investigations^20,41^.

Related to Thangam and Kathiresan^42^ classification, plant extracts are considered effective larvicides with LC_50_ < 100 mg/L, while extracts with LC_50_ > 200 mg/L are considered ineffective larvicides. Given this classification, the methylene chloride extract of *J. rubens* and the acetone extract of *C. sinuosa* can be categorized as effective for larvicidal biocontrol, as indicated by their respective LC_50_ values.

Furthermore, the larvicidal activity of the test macroalgae may be attributed to biochemical elements such as phenolics, terpenoids, flavonoids, saponins, tannins, and alkaloids, that may contribute to larvicidal action against *C. pipiens* larvae in combination or individually. Acheuk et al.^43^ showed that tannins have larvicidal and repellant effects that influence various phytophagous insects’ growth, development, and fecundity. According to González-Castro et al.^44^ and El-Saadony et al.^45^, terpenes, phenols, saponins, tannins, alkaloids, flavonoids, and anthraquinones extracted from macroalgae have larvicidal and repellant properties. Rashwan and Hammad^46^ documented the larvicidal activity of phenolic compounds from *A. platensis*.

The GC-MS analysis results of *C. sinuosa* acetone extract revealed that hexadecanoic acid methyl ester was about 41.3%. Besides, phthalic acid, butyl tetradecyl ester, and Bis (2- ethyl hexyl) phthalate were found only in *C. sinuosa* extract and strongly correlated LC_50_ and LC_90_ as recorded in PCA and scatter matrix with heatmap. Similarly, Javed et al.^47^ investigated the larvicidal activity of Bis-(2- ethylhexyl) phthalate produced by *Lactiplantibacillus plantarum* BCH 1. against *Culex quinquefasciatus* Say larva and 100% death was obtained at 250 ppm after exposure of 72 h, with LC_50_ value of 67.03 ppm. Arumugam et al.^48^followed as well, suggesting that the strong larvicidal activity of *Portieria hornemannii* (Rhizophyllidaceae) methanolic extracts was related to their high n-hexadecanoic acid concentration. Additionally, the presence of neophytadiene, 9-octadecenoic acid, hexadecanoic acid methyl ester and isochapin B among the phytochemicals identified in the methylene chloride extract of *J. rubens* might be responsible for the lethality shown by the extract against the mosquito larva^40,49,50^.

Also, the GC/MS analysis of *J. rubens* methylene chloride extract proved that the chemical with 44.52% content was n-hexadecanoic acid, which was highly related to alkaloids and flavonoids, as corroborated by heatmap correlation and PCA. Among the phytochemicals, hexadecanoic acid methyl ester is recognized as a fatty acid ester with antioxidant, antibacterial, and larvicidal properties^51^. Unlike green and brown algae, red algae are high in polyphenols and terpenes, which can disrupt the protein responsible for cholesterol transfer in larval growth, resulting in the death of larvae^52^.

According to the cell plot, the methylene chloride extract of the red algae *J. rubens* had the maximum T. phenols, alkaloids and saponins. This observation was further supported by PCA scatter plot, where the vectors representing T. phenols and T. terpenoids formed an acute angle with each other, as well as with 2-pentadecanone and 9-octadecenoic acid (Z), indicating a strong positive correlation among these parameters. The scatter plot and heatmap analyses further confirmed a positive correlation between T. terpenoids and all other parameters studied. This correlation suggests that terpenoids play a significant role in the bioactivity of algae extract. The toxic effects of algae extracts on mosquito larvae have been attributed to various mechanisms, including damage to the larvae’s gut caused by algae metabolites, reduction in larval feeding, inhibition of ATPase production, inhibition of digestive enzymes, and limitation of detoxification enzymes, as documented by Hassan et al.^53^.

Assessing the genotoxicity of algae extracts early in their action is of significant value. Therefore, we emphasize the utilization of the comet test in insect research to evaluate the genotoxic effects of various compounds and in ecological threat assessments. Most researchers have measured tail DNA (T DNA); however, some choose to measure the olive tail moment (OTM) and/or the TL. All these elements are presented to compare drug effects in future studies that use all three metrics^30^. Guanggang et al.^54^ studied all three elements to establish the mechanism of methomyl action. They discovered that 6 mM methomyl fragmented the DNA into big bits easily recognized and classified as TDNA by software. Likewise, *Curcuma longa* (Zingiberaceae) and *Melia azedarach* (Meliaceae) extracts produced DNA damage in *Culex quinquefasciatus* larvae^55^. Furthermore, *Achyranthes aspera* (Amaranthaceae) has been found to cause considerable DNA damage in *Aedes aegypti*^56^.

The current study found that mosquitos fed with *J. rubens* methylene chloride extract had the highest values of three metrics, TL, TDNA, and OTM, compared to positive control. This observation is consistent with earlier studies that have shown the usefulness of the comet assay as a quick and efficient technique for measuring DNA damage, with tail moment (TM) serving as a critical metric^57^. Furthermore, TM has the advantage of treating DNA damage as either a short tail with high division or a long tail with low division^58^. The consistency in the effects of algal extracts on DNA damage, as determined by the comet assay, highlights the relevance of the reported results. Specifically, the methylene chloride extract of *J. rubens* recorded the highest values for DNA damage and comet assay metrics, which could be attributed to the extract’s high concentration of saponins and phenols, as revealed by principal component analysis (PCA). According to Zulhussnain et al.^12^ genotoxicity cannot explain larval mortality in *Culex quinquefasciatus*. Still, it could be related to the presence of phenolic phytochemicals such as flavonoids, which change enzyme activity and hence cause *Culex quinquefasciatus* larvae death.

In the same trend, Raguvaran et al.^59^ concluded that under laboratory conditions, (2-2-ethyl-2 methylhexyloxycarbonyl)benzoic acid from *Bacillus pumilus* can cause DNA damage in a dose-dependent manner against *Aedes aegypti* larvae, *Anopheles stephensi*, and *Culex quinquefasciatus*. Kaur et al.^60^ reported that *Schizophyllum commune* (Schizophyllaceae) affecting *Spodoptera litura*, causing insect cell destruction and DNA strand breakages. Most toxic substances that cause DNA damage have been observed to produce a large amount of ROS and electrophilic free radicals. When natural products react with DNA, the cellular redox balance is disrupted, resulting in lipid and protein oxidation and DNA damage^61^.

## Methods

### Solvents and chemicals

Methanol (99.8%), acetone (99.9%), and methylene chloride (99.8%) were bought from Sigma-Aldrich (St. Louis, MO, USA). Egypt’s El Gomhouria Company for Trading Chemicals and Medical Appliances supplied all other analytical-grade chemicals.

### Macroalgae samples

*Jania rubens* (Linnaeus) J.V.Lamouroux (Rhodophyta: Corallinaceae) and *Coplomenia sinuosa* (Mertens ex Roth) Derbès & Solier (Heterokontophyta: Scytosiphonaceae) were collected from Alexandria, Egypt (Abo Qir 31°17′19″N, 30°00′57″E) during the summer of 2022. Specimen identification was conducted by Prof. Dr. Mostafa M. El-Sheekh of the Botany Department, Faculty of Science, Tanta University according to Aleem^62^ and Jha et al.^63^. The identification was also checked according to the recent information on https://www.algaebase.org/^64^. Voucher specimens (*Jania rubens*-Herb No. MU011 and *Colpomenia sinuosa*-Herb No. MU012) were deposited at the Botany Department’s herbarium at Mansoura University, Egypt. The algae samples were washed with seawater to remove sand and epiphytes before being cleaned with tap water. Afterward, 150 g fresh weight of each alga was air-dried in the shade for a week before being milled for extraction.

### Extract preparation

To improve the extraction efficiency, dried algae samples were ground to 60-mesh size sieve using electric blender (Moulenix, France) and the fine powder was taken for extraction. Then three different extracts, methanol (99.8%), acetone (99.9%), and methylene chloride (99.8%) for each alga were prepared separately in three different flasks by macerating 20 g fine algal powder in 200 mL solvent. The extraction process lasted for 48 h at room temperature in dark with continuous shaking at 200 rpm. Following extraction, the extracts were filtered through Whatman filter paper No. 1, concentrated by evaporation in a rotary vacuum evaporator and weighed. The crude extracts were resuspended in dimethyl sulfoxide (DMSO 99.7%) and kept at 4 °C for further use.

### Quantitative analyses of phytochemical constituents of the macroalgae extracts

#### Total phenolic content (TPC)

The total phenolic content was determined using the Folin-Ciocalteu reagent method published by Kamboj et al.^65^. Briefly, 0.5 mL of algae extract was mixed with 2.5 mL of Ciocalteu’s solution. The mixture was then diluted with 2.5 mL of Na_2_CO_3_ (7.5%) and left for 45 min at 45 °C. Each sample’s absorbance was calculated at 760 nm. TPC was expressed as mg gallic acid equivalent (GAE) per gram extract.

#### Total tannin content (TAC)

The total tannin content was calculated according to Mailoa et al.^66^ approach. Briefly, 1.0 mL of Folin-Ciocalteu reagent was mixed with 1.0 mL of algal extract and left at room temperature for 3 min. Then, 2 mL of 35% Na_2_CO_3_ was added, and the final volume was raised to 10 mL using dist. H_2_O. The mixture was properly mixed and then incubated for 30 min. The absorbance was measured at 725 nm, and the quantity was expressed as mg tannic acid equivalent (TAE) per gram of extract.

#### Total saponin content (TSC)

The vanillin-sulfuric acid assay technique was used to assess saponin concentration^67^. In a test tube, 200 µL of each algal extract was mixed with 5 µL of dist. water. The solvent was then dried in a water bath before adding 0.2 mL of freshly prepared vanillin-acetic acid (5%) solution and 1.2 mL of 70% perchloric acid then mixed thoroughly and incubated for 20 min at 70 °C. The tubes were cooled for 2 min before adding 5 mL of ethyl acetate. At 550 nm, the absorbance was compared to a blank and a standard.

#### Total alkaloid content (TAC)

About 3 mL of algal extract was mixed with 20 mL of 10% acetic acid/ethanol, sealed, and allowed to sit for four hours before being filtered and reduced to one-fourth of its original volume in a water bath. Concentrated ammonium hydroxide was put into each extract until it precipitated completely. The collected precipitate was then washed with mild ammonium hydroxide after it had settled and been filtered. Following 30 min of darkness, the absorbance at 512 nm was calculated. The alkaloid residue was dried and analyzed according to Harborne^68^. TAC was measured in mg of gallic acid equivalents (GAE) per gram extract.

#### Total flavonoid content (TFC)

Chang et al.^69^ described a colorimetric method for determining total flavonoid content (TFC). Briefly, 200 µL of each algal extract was combined with methanol (1.0 mL), 10% aluminum chloride (0.5 mL), dist.H_2_O (0.5 mL), and 1.0 M potassium acetate (0.5 mL). After 30 min of dark storage, the absorbance at 415 nm was measured. TFC was calculated using the standard curve and expressed in mg of quercetin equivalent (QCE) per gram extract.

#### Total terpenoid content (TTC)

The colorimetric assessment of total terpenoid concentration in each algal extract followed the methodology of Lukowski et al.^70^. Linalool was used as a standard for TTC determination in mg of linalool equivalents. All estimations were performed spectrophotometrically at 538 nm.

#### Metabolite chemical constituent analysis

A Thermo Scientific GC-TSQ mass spectrometer (Austin, TX, USA) and a direct capillary column TG-5 ms (30 m × 0.25 mm × 0.25 μm film thickness) were used for the Gas chromatography/Mass spectrometry (GC/MS) analysis of the extracts. The temperature of the column oven was initially set at 60 °C, then raised by 5 °C to 250 °C (held for 2 min), and then by 30 °C/min to 300 °C. The injection temperature was kept constant at 270 °C. Helium was used as a carrier gas, with a constant flow rate of 1.0 mL/min. After a 4 min solvent delay, the Autosampler AS3000 and split mode GC were used to inject 1 µL of the diluted samples. The mass spectrophotometric detector was operated in electron impact ionization mode with an ionizing energy of 70 e.v. scanning from the m/z range of 50–650. The ion source temperature was 230 °C. The electron multiplier (EM) voltage was maintained at 1250 V above autotune. The instrument was manually tuned using perfluorotributyl amine (PFTBA). The chemical components of the extracts were identified based on the mass spectral database of the NIST library.

#### Macroalgal extracts biopotential as mosquito larvicides

##### Mosquito culture

The larvae of *Culex pipiens* Linnaeus (Diptera: Culicidae) were collected from stagnant water sites surrounding Mansoura city, Egypt, using a rounded net dipper (20 cm in diameter) with a stainless-steel handle (150 cm in length) during the mid-spring of 2022. The larvae were then morphologically identified in the mosquito lab at the Economic Entomology Department, Faculty of Agriculture, Mansoura University, Egypt using previous keys described by^71^.

*Culex pipiens* colonies were maintained under controlled conditions at 29 °C, 85 ± 5% humidity, and a 16:8 h light/dark regime, following the method outlined by Farag^72^. Egg rafts were gently collected in 40 cm diameter bowls filled halfway with water. Upon hatching, neonates were fed a mixture of dried brewer’s yeast and ground-dried bread in a 1:2 ratio, increasing as larvae aged. Water was changed twice weekly, and larvae were monitored until pupation. Pupae were transferred to plastic pots and placed in wooden cages covered with muslin and a cloth sleeve. Upon emergence, adults were given oviposition bowls containing 1/5 tap water and Petri dishes lined with sponges soaked in a 10% sugar solution. Female mosquitoes were fed twice weekly with pigeon blood using a membrane-feeding system. Blood-fed females laid eggs in the oviposition bowls, where water temperature ranged from 23 to 30 °C. Mosquitoes were reared in the insectary for 10 generations before experimentation. Third-instar larvae of *C. pipiens* (5.21 ± 0.11 mg) were used for toxicity testing.

#### Larvicidal activity

The WHO^73^ approach assessed the larvicidal efficacy of methanol, acetone, and methylene chloride extracts from *J. rubens* and *C. sinuosa* against *C. pipiens* larvae in their early third instar. The bioassay involved randomly selecting groups of 25 *C. pipiens* larvae of the specified size and stage. Each group was placed in small disposable plastic cups (10 cm diameter and 5.5 cm height) containing 100 mL water. Five concentrations (50, 100, 150, 200 and 250 ppm) from each algae extract was prepared in DMSO (99.7%) and assessed for their larvicidal activity by applying 1 mL from each concentration to the larvae in the water. Except for the *J. rubens* methylene chloride extract, which was tested at 10 to 50 ppm concentrations. A control group was performed with each treatment using 1 mL DMSO (99.7%) to monitor the effect of the solvent on larvae. Also, a group containing water only (Blank) was run in parallel. Three replicates were prepared for each treatment. Mortality was measured at 24, 48, and 72 h following treatment, and the larval mortality percentages for each treatment concentration were computed following^74^ equation:

$$\:\text{M}\text{o}\text{r}\text{t}\text{a}\text{l}\text{i}\text{t}\text{y}\:\text{\%}=\frac{\text{X}-\text{Y}}{\text{X}}\:\times\:100$$

Where X is percentage survival of the control group and Y is that of the treated group. Also, fatal concentrations (LC_25_, LC_50_, and LC_90_) were calculated using probit analysis^75^ and a statistics program (LDP-line).

#### Ethical clearance

This experiment was authenticated by the members of the Ethics Committee of the Faculty of Science, Mansoura University, for Experiments on animals, their products, or genetic material, no. Sci-B-P-2024-246. The experimental protocol and the approval letter were added to the Supplementary material.

#### Molecular genotoxicity: alkaline single cell gel (SCG) assay

##### Comet assay sample preparation

For each sample, the entire body of five mosquitoes of late third larval instars of *C. pipiens* was combined with 200 µL of phosphate-buffered saline (PBS) (HiMedia TS1006) at pH 7.4.

#### Alkaline single cell gel (SCG) assay

Among the various extracts from *J. rubens* and *C. sinuosa*, the methylene chloride extract from *J. rubens* and the acetone extract from *C. sinuosa* were the most lethal to *C. pipiens* larvae in their third instar. As a result, they were chosen for further investigation into their genotoxicity in insects.

To evaluate the genotoxic effects of the methylene chloride extract from *J. rubens* and acetone extract from *C. sinuosa*, alongside negative control (DMSO) (Qualigens, CPW59) and positive control (Malathion 5), DNA damage was assessed in *C. pipiens* whole-body cells following the method described by Singh et al.^76^. At 1000 rpm, 20 µL of treated tissue solution from a pool of five insects was centrifuged for 10 min. Isolated hemocytes were suspended immediately in 50 µL of cold Ringer solution separated cells (10 µL) were mixed with 90 µL of 1% low melting point agarose (LMPA) and placed on microscope slides coated with 1.5% NMA. After adding a cover slip, the slides were immediately placed on ice. After the agarose had dried, the slides were submerged in a lysis solution (100 mM EDTA, 1% TritonX-100 (HiMedia, RM 845), 2.5 M NaCl, 0.25 M NaOH, 10 mM Tris, and 10% DMSO, pH = 10.0) for 24 h at 4 °C. The slides were submerged in a horizontal gel electrophoresis tank for 20 min following the lysis. That was filled with electrophoresis buffer containing 1 mM EDTA and 300 mM NaOH (pH = 13). Electrophoresis was carried out for 20 min at 270 mA and 24 V at 4 °C. The slides were neutralized with Tris-HCl (0.4 M, pH = 7.4), fixed in methanol, and dried overnight before being stained with 2 µg/mL EtBr. The comet was observed using an Axio fluorescence microscope (Carl Zeiss, Germany) with a 605 nm barrier filter and a 524 nm excitation filter. Three replicas were created, each with five individuals of the insect.

#### DNA damage assessment

EtBr-stained DNA was examined under a fluorescence microscope with a 40 X objective to observe DNA damage. Although various image analysis systems can quantify SCGE data, we used Kinetic Imaging’s Komet 5 image analysis software (Ltd., Liverpool, UK) with a CCD camera. This setup determined the quantitative and qualitative severity of DNA damage in cells by measuring the length of DNA migration and the percentage of moved DNA. The software then computed the tail moment. Each sample contained an average of 50 to 100 randomly selected cells. The migration rate per cell, the number of cells traveling faster, the extent of migration among injured cells, and cell viability were all evaluated.

#### Data analysis

Using a two-way ANOVA and Tukey’s multiple comparison test, the phytochemical data were analyzed using GraphPad Prism 9 (GraphPad Software, Inc., San Diego, CA, USA). Results were presented as means ± SD, with P-values < 0.05 indicating significant differences. All analyses were conducted in triplicate. Probit analysis (Finney, 1971) was used to predict lethal concentrations (LC_25_, LC_50_, and LC_90_) using a statistics program (LDP-line) with 95% fiducial bounds on upper and lower confidence limits, Chi-square, slope, standard error, and confidence intervals. One-way ANOVA was used to test the significance of larval mortality percentages, followed by Duncan’s multiple range test *post hoc*. The statistical analysis was conducted using IBM SPSS Statistics for Windows, Version 23.0, with a significant level of α = 0.05. Comet data characteristics, such as tail length (TL), tail DNA % (T DNA), and olive tail moment (OTM), were analyzed using one-way ANOVA in GraphPad Prism 9. Three replicates of each treatment represented the comet data. JMP^®^, Version 17.2.0 (SAS Institute Inc., Cary, NC, USA, 2022–2023) was used to create a cell plot of all analyzed parameters in response to the *C. sinuosa* and *J. rubens* extracts. JMP^®^, Version 17.2.0 was employed to estimate the intercorrelations (scatter plot, heatmap correlations, principal component analysis (PCA), and biplot) between different phytochemical parameters and some selected metabolites of GC/MS analysis of the acetone extract from *C. sinuosa* and the methylene chloride extract from *J. rubens*, as well as their larvicidal and Genotoxicity activities on late 3rd larval instars of *C. pipiens*.

## Conclusion

It can be concluded that the methylene chloride extract from *J. rubens* and the acetone extract from *C. sinuosa* are effective larvicides against *Culex pipiens* third instar larva with low LC_50_ values. The methylene chloride extract from *J. rubens* is the most toxic, maintaining 100% mortality of *C. pipiens* at 50 ppm after 72 h. GC/MS analysis of these extracts confirmed the presence of phytochemicals known to have larvicidal activity, further validating their efficacy as larvicides. Assessing the genotoxicity of these extracts in insect species post-exposure to different toxins, including biopesticides, presents a promising avenue for future research. Such tests provide insights into the biology of the targeted insect species and contribute valuable data for developing ecologically friendly larvicides that are effective against *C. pipiens.* However, despite the promising results, further research is warranted to develop novel insecticides that address concerns related to eco-compatibility and market acceptance in the pesticide manufacturing industry.

## Electronic supplementary material

Below is the link to the electronic supplementary material.

Supplementary Material 1

## Data Availability

All data generated or analyzed during this study are included in this published article [and its supplementary information files].
