# Supplementary material for: Characterization of some selected macroalgae extracts and assessment of their insecticidal and genotoxicity in Culex pipiens L. mosquito larvae
Source: Sci Rep. 2025 Jan 21;15:2655. doi: 10.1038/s41598-025-86347-7 (PMC11751296; doi:10.1038/s41598-025-86347-7)
Supplement: Supplementary file 1 — Supplementary Material 1 [file 41598_2025_86347_MOESM1_ESM.docx]

**Experimental Protocol**

**1- General information**

| **Protocol title** | |
| --- | --- |
| **English** | Characterization of some selected macroalgae extracts and assessment of their insecticidal and genotoxicity in *Culex pipiens* L. mosquito larvae |

| **Name and title of the investigator**(s) who is (are) responsible for conducting the research, and the address and telephone number(s) of the research site(s), including responsibilities of each. | | | | |
| --- | --- | --- | --- | --- |
| **E-mail** | **Phone number** | **His / Her role in the study** | **Department** | **Principle Investigator Name** |
| [dalirefy@mans.edu.eg](mailto:dalirefy@mans.edu.eg) | 01000345478 | Designed the whole study, conducted the experiments, interpreted the results, writing, editing, and formatting of the manuscript. | Botany Department, Faculty of Science, Mansoura University | **Dina Ali Refaay Ali** |
| **E-mail** | **Phone Number** | **His / Her role in the study** | **Department** | **Co-Author Name** |
| [mostafaelsheikh@science.tanta.edu.eg](mailto:mostafaelsheikh@science.tanta.edu.eg) | +201224106666 | Designed the whole study, writing, editing, and formatting of the manuscript | Botany Department, Faculty of Science, Tanta University | [**Mostafa M. El-Sheekh**](https://orcid.org/0000-0002-2298-6312) |
| [Yasminheikal@mans.edu.eg](mailto:Yasminheikal@mans.edu.eg) |  | Designed the whole study, conducted the experiments, conducted the analysis and visualization of data, interpreted the results and writing, editing, and formatting of the manuscript | Botany Department, Faculty of Science, Mansoura University | **Yasmin M. Heikal** |
| [arabdel@mans.edu.eg](mailto:arabdel@mans.edu.eg) |  | Designed the whole study, conducted the experiments, interpreted the results and writing, editing, and formatting of the manuscript | Economic Entomology Department, Faculty of Agriculture, Mansoura University | **Ahmed A. Rashed** |

1. **Name and address** of the sponsor/funder.

This work was funded by Princess Nourah bint Abdulrahman University Riyadh, Saudi Arabia, by grant No. PNURSP2024R182.

1. **Name and address where the research will be conducted**

Botany Department, Faculty of Science and Economic Entomology Department Faculty of Agriculture in Mansoura University

1. **Name(s) and address**(es) of the clinical laboratory(ies) and other medical and/or technical department(s) and/or institutions involved in the research. (If any)

Botany Department, Faculty of Science and Economic Entomology Department Faculty of Agriculture in Mansoura University

**2-Introduction:**

**Background:**

Macroalgae are regarded one of the most significant biomass producers in the marine environment. They synthesize an extensive variety of structurally distinct secondary metabolites with pharmacological and biological functions, the reason they have long been associated with human life and widely used as a source of food, feed, fertilizer, and medicine [1, 2]. Despite the variation in macroalgae pigmentation, they also differ in their metabolic features [3]. The phytochemical compounds significantly differ among macroalgae according to environmental conditions (salinity, light intensity and temperature), as well as genetic changes across species [4].

1. **Literature review**/current state of knowledge about project topic,

Macroalgae are rich in protein, polysaccharides, vitamins, minerals, fatty acids, and other bioactive substances such as proline, betaine, phenolics, flavonoids, and sterols with a vast range of biological activities like antimicrobial, antifouling, anticancer and antioxidants [5]. In addition, macroalgae produce carrageenan, algin and agar, which are employed as stabilizing and gelling agents [6]. Macroalgae also synthesize compounds with antibiotic actions including phloroglucinol, bromo-phenols, terpenoids, and tannins, which may serve as natural nematocidal and insecticidal activities [7].

Several macroalgae species are abundant along the Mediterranean coast of Alexandria (Egypt) characterized by their phytochemical constituents with various biological potentialities and larvicidal in particular. For instance, the phytochemical constituents of *Jania rubens* are rich in carotenoids, flavonoids, vitamins, diterpenes, phenolics, fatty acids, tannins, and several other secondary metabolites that possess cytotoxic, antimicrobial, anticancer, and molluscicide effects [8]; [9]; [10] ;[11]. Abdel Haleem et al. [12] documented the larvicidal effect of *Jania rubens* methanolic extract for *Culex pipiens* third instar larva control. Moreover, phenolic compounds, sterols, unsaturated fatty acids, and fucoxanthin from the brown alga *Colpomenia sinuosa* have antioxidant, anti-inflammatory, and antibacterial properties [13]. Similarly, [14] recorded the larvicidal effect of acetone extract from *Colpomenia sinuosa* against *Artemia salina.*

In the production of bioinsecticides from algae extracts, they are low cost, biodegradable and safe substitutes to chemical pesticides which are harmful for human health. They contain numerous active chemicals with possible pesticidal action, which can help with pest management and contribute to sustainable agriculture [15]. Several studies have referred to the significant role of macroalgae extracts in pest management such as bugs, moths, termites, and mosquitoes [16]; [17]; [18].

Detecting the effect of insecticides from algae on cell structure, DNA functions, and cell mutation of insects is crucial for determining the degree of potency of such biocontrol agents compared with that of synthetic ones [19]. In this regard, molecular biology approaches, such as comet assays, enable a novel tool to investigate ecological toxicity processes at the cellular and molecular levels. Whereas at alkaline pH, strand breakage and alkali labile sites can be measured, allowing for a more sensitive and integrated approach to measuring genotoxicity [20]. Because of its multiple benefits, it is commonly used in medical research and ecotoxicology. The comet test can be used to examine the genotoxic compounds mode of action, damage in DNA induced by oxidative stress, environmental pollution as well as using sentinel organisms [21]. The comet test has become a tool for investigating interactions between diverse xenobiotics. It is particularly valuable for studying insecticide resistance, the evolution of environmental pollutant immunity, and better understanding insect ageing. Currently, the comet assay is frequently used to analyze terrestrial varieties of numerous categories, like insects which are important in human economy [22].

Employing various extraction methods and solvents can increase the amount of bioactive chemicals from macroalgae biomass [23]. Solid-liquid extraction is the most often used extraction process for obtaining phytochemicals from diverse plants, including macroalgae, due to its simplicity, low cost, and time efficiency. Several studies referred to solvents with different polarities such as acetone, ethanol, and methanol to ensure maximum extraction yield [24]; [25].

1. Justification for study

Replacing chemical insecticides with algae-based bioinsecticides against *C. pipiens* mosquitos.

1. **Intended/potential** use of study findings

Macroalgae extracts might be recommended as ecologically friendly larvicides that are also genocidal against *C. pipiens*.

1. Objectives: Clearly describe the primary and any secondary objectives of the study, or specific hypotheses being tested.

The present work aiming to i) study the effect of different solvent extraction on the composition of phytochemicals in *J. rubens* and *C. sinuosa,* ii) investigate the potential of the different extracts as larvicide against *Culex pipiens* iii) evaluate the molecular genotoxicity of the promising extracts by comet assay and iv) estimate the correlation among different assays.

1. **Hypotheses or questions**
2. **Aim of the work**

Therefore, the present work originated aiming to i) study the effect of different solvent extraction on the composition of phytochemicals in *J. rubens* and *C. sinuosa,* ii) investigate the potential of the different extracts as larvicide against *Culex pipiens* iii) evaluate the molecular genotoxicity of the promising extracts by comet assay and iv) estimate the correlation among different assays.

3- Methods

1. **Study design:** For each experiment, give brief details of the study design:

Time-line diagrams or flow chart can be useful to illustrate how complex study designs were carried out.


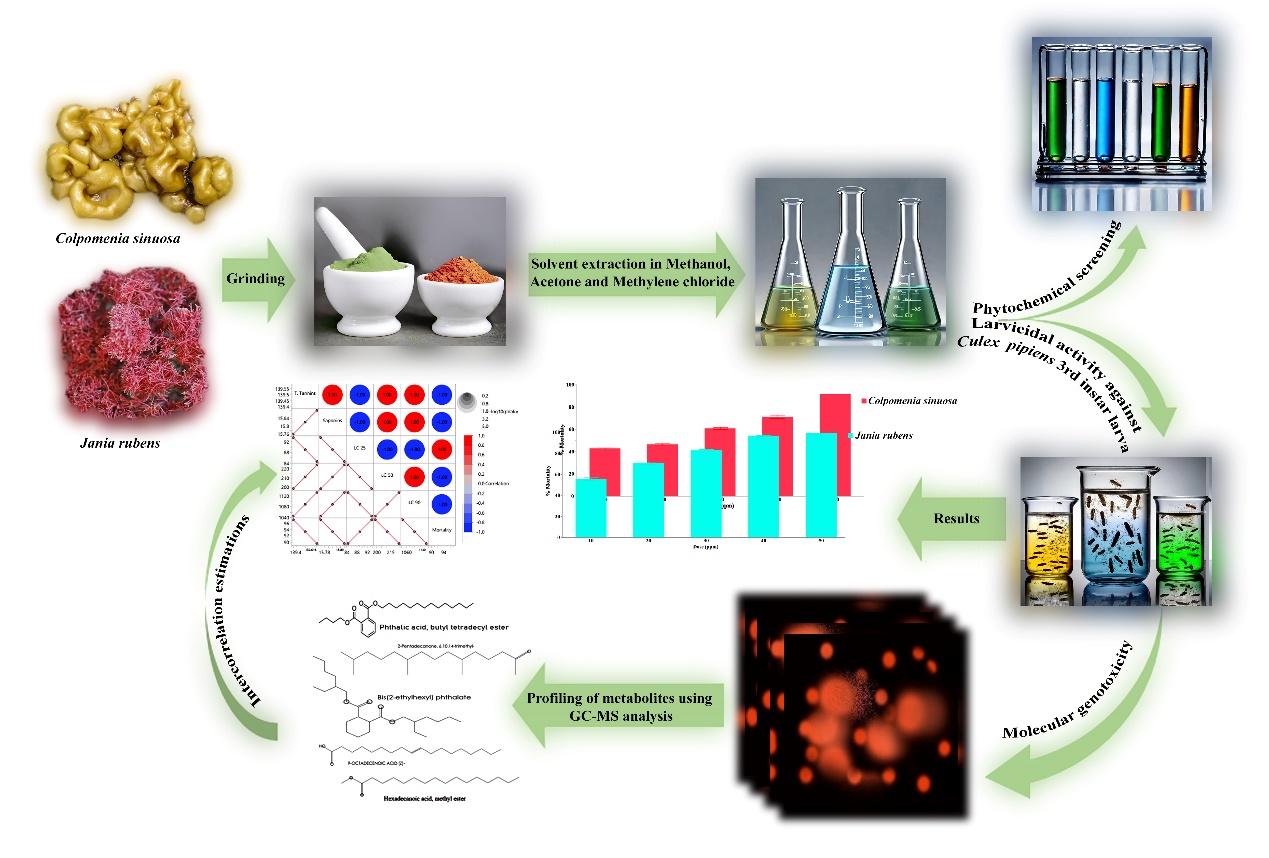


1. Experimental procedures: For each experiment and each experimental group, including controls, provide precise details of all procedures carried out.

***Macroalgae samples***

*Jania rubens* (Linnaeus) Lamouroux (Corallinaceae) and *Coplomenia sinuosa* (Mertens ex Roth) Derbès & Solier (Scytosiphonaceae) were collected from Alexandria, Egypt (Abo Qir [31°17′19″N, 30°00′57″E](https://tools.wmflabs.org/geohack/geohack.php?language=ar&pagename=%D9%82%D8%B5%D8%B1_%D8%A7%D9%84%D9%85%D9%86%D8%AA%D8%B2%D9%87&params=31.2886_N_30.0159_E_globe:earth)) in summer, 2022. Identification of specimens were made by Prof. Dr. [Mostafa M. El-Sheekh](https://orcid.org/0000-0002-2298-6312) (Botany Department, Faculty of Science, Tanta University). Voucher specimens (*Jania rubens*-Herb No. MU011 and *Coplomenia sinuosa*-Herb No. MU012) were deposited at the herbarium of Botany Department, Faculty of Science, Mansoura University, Egypt. The Algae samples were washed with seawater to get rid of sand particles and epiphytes before being cleaned with tap water then left to air dried in the shade and milled for subsequent extraction.

***Extract preparation***

Extraction was performed using a distinct solvent system. The weight of 20 g powdered biomass of each test alga was soaked in methanol, acetone, and methylene chloride separately. The extraction continued for 48 h with continuous shaking at 200 rpm in room temperature. The extracts were then dried using a rotary evaporator before being weighed and kept at 4 ° C for future use.

***Quantitative analyses of phytochemicals constituents of the macroalgae extracts***

***Total phenolic content (TPC)***

The total phenolic content has been evaluated by the Folin-Ciocalteu reagent method described by Kamboj et al. [26]. Gallic acid standard solutions (100-200 g/mL) were used for the standard calibration curve preparation. For each algae extract, 0.5 mL was combined with 2.5 mL of Ciocalteu's solution. Afterward, the mixture was further diluted using 2.5 ml of Na_2_CO_3_ (7.5 %) and left for 45 minutes at 45 °C. For each sample the absorbance was computed at 760 nm. Standard curve was used to calculate TPC that was presented as mg gallic acid equivalent (GAE) per g extract.

***Total tannins content (TAC)***

The total tannin content was estimated using Mailoa et al. [27] technique, with some modifications. Briefly, Folin-Ciocalteu reagent (1.0 mL) was combined with algal extract (1.0 mL) then left at room temperature for 3 min. Next, 2 mL of 35% Na_2_CO_3_ was added, and the final volume was increased to 10 mL using dist. H_2_O. The mixture was thoroughly agitated, and incubated for 30 min. At 725 nm the absorbance was determined, and the concentration was evaluated related to the tannic acid standard curve and given as mg tannic acid equivalent (TAE) / g extract.

***Total saponins content (TSC)***

The saponin concentration of algal extracts was determined using the vanillin-sulfuric acid assay technique of Li et al. [28]. In brief, 2.4 mg/mL oleanolic acid stock solution was in standard curve preparation. In a test tube, 200 µL of each algal extract was combined with 5 µL of dist. water. The solvent was then dried in a water bath before adding 0.2 mL of freshly prepared vanillin-acetic acid (5 %) solution and 1.2 mL of 70 % perchloric acid, mixing thoroughly, and incubating for 20 minutes at 70 ° C. The tubes were then cooled for 2 minutes before 5 mL of ethyl acetate was added. At 550 nm, the absorbance was compared to a blank and a standard.

***Total alkaloid content (TAC)***

About 3 mL macroalgae extract was dropped in a 50-mL beaker, followed by 20 mL of 10% acetic acid/ethanol. The solution was sealed and left to sit for four hours before filtration and reduction to the fourth of the original volume in a water bath. To each extract, concentrated ammonium hydroxide was dropped until complete precipitation. Then weak ammonium hydroxide was used for washing the collected precipitate that sifted once settled. After 30 minutes of darkness, the absorbance at 512 nm was estimated. Gallic acid served as the calibration reference. The alkaloid residue was dried and analyzed according to [29]. TAC was measured in mg of Gallic acid equivalents (GAE) per g extract.

***Total flavonoid content (TFC)***

[30] described a colorimetric method for determining total flavonoid content (TFC). Briefly, 200 µL of each algal extract was combined with methanol (1.0 mL), 10 % of aluminum chloride (0.5 mL), dist.H_2_O (0.5 mL), and 1.0 M potassium acetate (0.5 mL). Samples were then stored in the dark for 30 minutes and the absorbance at 415 nm was determined. TFC was determined related to the standard curve and expressed as mg of quercetin equivalent (QCE) per gramme extract.

***Total terpenoid content (TTC)***

The colorimetric assessment of total terpenoid concentration in each algal extract followed the methodology of [31]. Linalool was used as a standard for TTC determination in mg of linalool equivalents. All estimations were performed spectrophotometrically at 538 nm.

***Metabolite chemical constituent analysis***

A Thermo Scientific GC-TSQ mass spectrometer (Austin, TX, USA) and a direct capillary column TG-5 ms (30 m x 0.25 mm x 0.25 µm film thickness) were used for the GC/MS analysis of the extracts. The temperature of column oven was initially set at 60 °C, then raised by 5 °C to 250 °C (held for 2 min), and then by 30 °C/min to 300°C. The injection temperature kept constant at 270 °C. Helium was used as a carrier gas, with a constant flow rate of 1.0 mL/min. After a 4 min solvent delay, the Autosampler AS3000 and split mode GC were used to inject 1 µL of the diluted samples. The mass spectrophotometric detector was operated in electron impact ionization mode with an ionizing energy of 70 e.v. scanning from m/z range of 50–650. The ion source temperature was 230 °C. The electron multiplier voltage (EM voltage) was maintained at 1250 V above auto tune. The instrument was manually tuned using perfluorotributyl amine (PFTBA). The chemical components of the extracts were identified based on the mass spectral database of Wiley and Wiley NIST libraires.

***Macroalgal extracts' biopotential as mosquito larvicides: An eco-friendly strategy to controlling* *Culex pipiens***

**Mosquito culture:** the larvae of *Culex pipiens* were collected from stagnant water areas around Mansoura city, Egypt and identified in the Mosquito lab at Economic Entomology Department, Faculty of Agriculture, Mansoura University, Egypt. *Culex pipiens* colonies were maintained at 29 °C, 85 ± 5% humidity and 16:8 hr. light/dark. The rearing of *C. pipiens* was performed according to the method explained by [32] at the Mosquito lab at Economic Entomology Department, Faculty of Agriculture, Mansoura University, Egypt. Egg rafts were collected from the natural ponds, transferred to the lab, and gently gathered in bowls (40 cm in diam.) half filled with free-chlorine water. After enclosing, the neonates were fed with a fine mixture of dried brewer's yeast and ground-dried bread in a ratio of 1:2, respectively. The amount of this mixture increased as the larval age progressed. Water was renewed twice a week and larvae were monitored in these pots until pupation. Pupae were separated into plastic pots and then transported to wooden cages covered with muslin on all sides and a cloth sleeve on one side until they emerged as adults. The cages of adults were provided with oviposition bowls filled with 1/5 tap water and Petri dishes lined with sponges saturated with a 10 % sugar solution, as a food for adults. Adult food was renewed daily after cleaning up both the petri dishes and the sponges. *Culex* females were deprived of sugar then provided with one pigeon for blood feeding twice a week during the research time. A single bird may be used once or twice a week for more than a year if so desired. A pigeon is placed in the cage and left overnight with mosquitoes. To obtain good feeding, sugar solution is removed from the cage at least 24 hours before the bird is placed in the cage. It is important to render the bird relatively immobile so that it will not frighten the mosquitoes away or kill them. This is accomplished by taping the bird in a cylindrical piece of hardwood cloth or upside down in an instrument pan (9 x 22 x 4 cm) after removing feathers from a sizeable area in the pectoral region. The immobilized bird, breast up, is placed in the cage and allowed to remain overnight. The following morning the bird is returned to its cage. A single bird may be used once or twice a week for more than a year if so desired. At the end of the experiment the bird was euthanized, and the dead bird was disposed of hygienically. Next, blood-feeding females were ready to lay their eggs on the water surface in the oviposition bowls where water temperature ranged between 23 - 30 °C.

***Larvicidal activity***

The [33] approach was used to assess the larvicidal efficacy of methanol, acetone, and methylene chloride extracts from *J. rubens* and *C. sinuosa* against *C. pipiens* larvae in their early third instar at the Mosquito lab at Economic Entomology Department, Faculty of Agriculture, Mansoura University, Egypt. Except for the methylene chloride extract from *J. rubens*, which was studied in the range of 10 to 50 ppm, each extract was placed in DMSO for graded series of concentrations (50 to 250 ppm) preparation. At 24, 48, and 72 hours after treatment, mortality was recorded. For toxicological testing, batches of 25 third instar larva of *C. pipiens* were transported to small disposable plastic cups (diameter 10 cm, height 5.5 cm) holding five different doses of each algal extract prepared in 100 mL of free chlorine water. For each concentration and control, three replicates were run. The larval mortality percentages were computed for each treatment concentration. The mortality results were revised in accordance with [34].

***Molecular Genotoxicity:*** ***alkaline Single Cell Gel (SCG) assay***

***Comet assay sample preparation***

For each sample, the entire body of five mosquitoes of late third larval instars of *C. pipiens* was combined with 200 µL of phosphate buffered saline (PBS).

***Alkaline Single Cell Gel (SCG) assay***

It's noteworthy to point out that among the different extracts from *J. rubens* and *C. sinuosa*, the methylene chloride extract from *J. rubens* and acetone extract from *C. sinuosa* was the most toxic against the 3^rd^ instar larvae of *C. pipiens* and therefore they were selected to follow their genotoxicity in the insect.

To assess the genotoxic effects of the methylene chloride extract from *J. rubens* and acetone extract from *C. sinuosa* compared with the negative control (DMSO) and the positive control (Malathion 5), DNA damage was determined in *C. pipiens* whole-body cells according to Singh [20]. At 1000 rpm, 20 µL treated tissue solution from a pool of five insects was centrifuged for 10 minutes. Isolated hemocytes were suspended immediately in 50 µL of cold Ringer solution. Separated cells (10 µL) were combined with 90 µL of 1% low melting point agarose (LMPA) and placed on microscope slides coated with 1.5% NMA. Slides were placed on ice immediately after adding a cover slip. Slides were immersed in a lysis solution (100 mM EDTA, 1% TritonX-100, 2.5 M NaCl, 0.25 M NaOH, 10 mM Tris, and 10 % DMSO, pH = 10.0) after the agarose had dried out, at 4 °C for 24 hours. The slides were immersed in a horizontal gel electrophoresis tank after lysis for 20 min. that was filled by electrophoresis buffer containing 1 mM EDTA and 300 mM NaOH (pH=13). Electrophoresis was performed for 20 minutes at 270 mA and 24 V at 4 ° C. In Tris-HCl (0.4 M, pH 7.4) the slides were neutralized, fixed in methanol then dried overnight before staining with 2 µg/mL ethidium bromide (EtBr). An Axio fluorescence microscope (Carl Zeiss, Germany) equipped with a 605 nm barrier filter and a 524 nm excitation filter was used for comets observation. Three replicas were created, each with a group of five individuals.

***DNA Damage Assessment***

To observe the DNA damage, EtBr-stained DNA is examined under a fluorescence microscope with a 40X objective (based on the dimensions of the cells being counted). Although any image analysis system could be used to quantify SCGE data, we used Kinetic Imaging's Komet 5 image analysis software (Ltd., Liverpool, UK) in conjunction with a CCD camera to assess the quantitative and qualitative severity of DNA damage in cells by measuring the length of DNA migration and the percentage of migrated DNA. Finally, the program calculated the tail moment. On average, 50 to 100 randomly selected cells are analyzed every sample. Compare the migration rate per cell, the number of cells that move faster, the extent of migration among damaged cells, and viability.

1. Experimental animals: (For protocols using experimental animals)

- Genus: *Culex*
- Species*: pipiens*
- The experiments were achieved on 3^rd^ instar larvae at Economic Entomology Dept., Faculty of Agriculture, Mansoura University.
- Source of insects: Mosquito lab at Economic Entomology Dept., Faculty of Agriculture, Mansoura University.

1. Housing and husbandry

**1. Housing**

*Culex pipiens* larvae thrive in such containers of stagnant water, the more polluted with organic material, the better.

**2. Husbandry conditions**

*Culex pipiens* colonies were maintained at 29 °C which seems to be the optimal temperature for growth. Egg rafts were collected from the natural ponds, transferred to the lab, and gently gathered in bowls (40 cm in diam.) half filled with free-chlorine water. After enclosing, the neonates were fed with a fine mixture of dried brewer's yeast and ground-dried bread. Water was renewed twice a week, and larvae were monitored in these pots until pupation. The cages of adults were provided with oviposition bowls filled with 1/5 tap water and Petri dishes lined with sponges saturated with a 10 % sugar solution, as a food for adults. The water temperature ranged between 23 - 30 °C.

3-Sample size

For toxicological tests, batches of 25 *C. pipiens* 3^rd^ instar larvae were transferred to small disposable plastic cups (diameter 10 cm, high 5.5 cm).

4- Allocating animals to experimental groups

a. Give full details of how animals will be allocated to experimental groups, including randomization or matching

5-Statistical methods

Data of the phytochemical analyses were analyzed using GraphPad Prism 9 with two-way ANOVA and Tukey's multiple comparison test. The results were given as means ± SD. P-values < 0.05 showed significant differences. All analyses were conducted in triplicate. Using Probit analysis [35], the lethal concentrations (LC_25_, LC_50_, and LC_90_) were calculated and a statistics program (LDP-line), with 95% fiducial bounds on upper and lower confidence limits, Chi-square, slope, standard error, and confidence intervals. Using one-way ANOVA, larval mortality percentages were assessed for significance, followed by the post-hoc Duncan's multiple range test. Using IBM SPSS Statistics for Windows, Version 23.0, the statical analysis was performed. The significance level was set to α = 0.05. The data parameters of comets including tail length (TL), tail DNA percentage (T DNA), and olive tail moment (OTM) were analyzed using one-way ANOVA in GraphPad Prism 9 (GraphPad Software, Inc., San Diego, CA, USA). Comet data was represented by three replicates of each treatment. JMP®, Version 17.2.0 (SAS Institute Inc., Cary, NC, USA, 2022-2023) was used to create a cell plot of all analyzed parameters in response to the C. sinuosa and J. rubens extracts. JMP®, Version 17.2.0 was employed to estimate the intercorrelations (scatter plot, heatmap correlations, principal component analysis (PCA), and biplot) between different phytochemical parameters and some selected metabolites of GC/MS analysis of the acetone extract from *C. sinuosa* and the methylene chloride extract from *J. rubens*, as well as their larvicidal and genotoxicity activities on late 3^rd^ larval instars of *C. pipiens*.

**d)** Generalizability/ translation

Not applicable generally.

Funding

This work was funded by Princess Nourah bint Abdulrahman University Riyadh, Saudi Arabia, by grant No. PNURSP2024R182.

1. Safety Considerations

The safety of research participants is foremost. Safety aspects of the research should always be kept in mind and information provided in the protocol on how the safety of research participants will be ensured. This can include procedures for recording and reporting adverse events and their follow-up, for example. It is useful to remember that even administering a research questionnaire can have adverse effects on individuals.

**All safety and precautions are taken into consideration by all authors.**

1. Dissemination of Results and Publication Policy
2. References:

1. Ganesan, A.R., Tiwari, U., and Rajauria, G. Seaweed nutraceuticals and their therapeutic role in disease prevention*.* *Food Sci Hum Wellness*, **8**(3): p. 252-263 (2019).

2. Dobrincic, A., et al. Advanced Technologies for the Extraction of Marine Brown Algal Polysaccharides*.* *Mar Drugs*. **18**(3): p. 168 (2020).

3. Chakraborty, K., Joseph, D., and Praveen, N.K. Antioxidant activities and phenolic contents of three red seaweeds (Division: Rhodophyta) harvested from the Gulf of Mannar of Peninsular India. *J Food Sci Technol*. **52**(4): p. 1924-35 (2015).

4. Garcia-Vaquero, M., et al. Exploring Ultrasound, Microwave and Ultrasound-Microwave Assisted Extraction Technologies to Increase the Extraction of Bioactive Compounds and Antioxidants from Brown Macroalgae. *Mar Drugs*. **18**(3): p. 172 (2020).

5. Suraiya, S., et al. Monascus spp. fermented brown seaweeds extracts enhance bio-functional activities*. Food biosci*. **21**: p. 90-99 (2018).

6. Peñalver, R., et al. Seaweeds as a functional ingredient for a healthy diet. *Marine Drugs*. **18**(6): p. 301 (2020).

7. Suganya, S., et al. New insecticides and antimicrobials derived from *Sargassum wightii* and *Halimeda gracillis* seaweeds: Toxicity against mosquito vectors and antibiofilm activity against microbial pathogens. *S Afr J Bot.* **125**: p. 466-480 (2019).

8. El-Din, S.M.M. and El-Ahwany, A.M. Bioactivity and phytochemical constituents of marine red seaweeds *(Jania rubens, Corallina mediterranea* and *Pterocladia capillacea).* *J Taibah Univ Sci*. **10**(4): p. 471-484 (2016).

9. Abdel-Raouf, N., et al. In vivo application of *Jania rubens* silver nanoparticles as a chemopreventive agent. *Aust. J. Basic Appl. Sci*. **11**: p. 176-186 (2017).

10. Gheda, S., El-Sheekh, M. and Abou-Zeid, A. In vitro anticancer activity of polysaccharide extracted from red alga *Jania rubens* against breast and colon cancer cell lines. *Asian Pac J Trop Med.* **11**(10): p. 583-589 (2018).

11. Hamid, S.S., et al. Metabolome profiling of various seaweed species discriminates between brown, red, and green algae*.* *Planta*. **249**: p. 1921-1947 (2019).

12. Abdel Haleem, D.R., et al. Screening and evaluation of different algal extracts and prospects for controlling the disease vector mosquito *Culex pipiens L.* *Saudi J Biol Sci*. **29**(2): p. 933-940 (2022).

13. Al Monla, R., et al. The Cytotoxic and Apoptotic Effects of the Brown Algae *Colpomenia sinuosa* are Mediated by the Generation of Reactive Oxygen Species*.* Molecules. **25**(8): p. 1993 (2020).

14. Udayan, E., et al. A case study of screening for larvicidal activity of *Ulva reticulata* and *Colpomenia sinuosa* acetone extracts against Artemia salina*.* *J. Algal Biomass Util*. **11**(1): p. 84-88 (2020).

15. Costa, J.A.V., et al. Potential of microalgae as biopesticides to contribute to sustainable agriculture and environmental development. *J Environ Sci Health*, Part B. **54**(5): p. 366-375 (2019).

16. Sahayaraj, K. and Jeeva, Y.M. Nymphicidal and ovipositional efficacy of seaweed *Sargassum tenerrimum (*J. Agardh*)* against *Dysdercus cingulatus (*Fab.*)(Pyrrhocoridae).* *Chil J Agri Res*. **72**(1): p. 152-156 (2012).

17. Salvador-Neto, O., et al. Larvicidal Potential of the Halogenated Sesquiterpene (+)-Obtusol, Isolated From the Alga *Laurencia dendroidea* J. Agardh (Ceramiales: Rhodomelaceae), against the Dengue Vector Mosquito *Aedes aegypti* (Linnaeus) (Diptera: Culicidae). *Mar Drugs*. **14**(2): p. 20 (2016).

18. Ishii, T., et al. Insecticidal and repellent activities of laurinterol from the Okinawan red alga *Laurencia nidifica.* *Rec Nat Prod*. **11**(1): p. 63-68 (2017).

19. Azqueta, A., Shaposhnikov, S., Collins, A. Detection of oxidised DNA using DNA repair enzymes, in: A. Dhawan, D.R. Anderson (Eds.), The Comet Assay in Toxicology, . The Comet Assay in Toxicology, RSC Publ., Cambridge: p. 57–78 (2009).

20. Singh, N.P., et al. A simple technique for quantitation of low levels of DNA damage in individual cells*.* *Exp Cell Res*. **175**(1): p. 184-91 (1988).

21. Ma, F.F., Carbone, F., Forti, G.C., Buschini, A., Poli, P., Rossi, C., Marabini, L., Radice, S., Chiesara, E. and Hrelia, P. Drinking water quality: An in vitro approach for the assessment of cytotoxic and genotoxic load in water sampled along distribution system. *Environ Int.* (35): p. 1053–1061(2009).

22. Augustyniak, M., Gladysz, M. and Dziewiecka, M. The Comet assay in insects--Status, prospects and benefits for science. *Mutat Res Rev Mutat Res*. **767**: p. 67-76 (2016).

23. Michalak, I. and Chojnacka, K. Algae as production systems of bioactive compounds. *Eng Life Sci*. **15**(2): p. 160-176 (2015).

24. López, A., et al. The effects of solvents on the phenolic contents and antioxidant activity of *Stypocaulon scoparium* algae extracts. *Food Chem*. **125**(3): p. 1104-1109 (2011).

25. Alassali, A., et al. Methods for Upstream Extraction and Chemical Characterization of Secondary Metabolites from Algae Biomass. *Adv techn biol med*. **4**: p. 1-16 (2016).

26. Kamboj, A., et al. Application and Analysis of the Follin Ciocalteu Method for the Determination of the Total Phenolic Content from Leaves, Stems and Seeds of *Cucumis sativus L. J Pharm Res*. **9**(5): p. 323-329 (2015).

27. Mailoa, M.N., et al. Tannin extract of guava leaves (*Psidium guajava* L) variation with concentration organic solvents. *Int J Sci Technol Res*. **2**(9): p. 106-110 (2013).

28. Li, J., et al. Optimization of microwave-assisted extraction of triterpene saponins from defatted residue of yellow horn (Xanthoceras sorbifolia Bunge.) kernel and evaluation of its antioxidant activity*.* *Innov Food Sci Emerg Technol.* **11**(4): p. 637-643 (2010).

29. Harborne, J.B. Phenolic compounds. Phytochemical methods. A guide to modern techniques of plant analysis: p. 33-88 (1973).

30. Chang, C.C., et al. Estimation of total flavonoid content in propolis by two complementary colorimetric methods*.* *J food drug anal*. **10**(3) (2002).

31. Lukowski, A., et al. Adaptation of a simple method to determine the total terpenoid content in needles of coniferous trees. *Plant Sci*. **314**: p. 111090 (2020).

32. Farag, S., El-Sayed, A. and Abdel-Haleem D. Larvicidal Efficacy of Nigella Sativa Seeds Oil and It’s Nanoparticles against *Culex Pipiens* and *Musca Domestica*. *J Egypt Soci Parasitol.* **50**(1): p. 215-220 (2020).

33. WHO, Guidelines for laboratory & field testing of mosquito larvicides. Bull.World Health Org., p. 1-4 (2005).

34. Abbott, W.S. A Method of Computing the Effectiveness of an Insecticide*.* *J Econom Entomol.* **18**(2): p. 265-267 (1952).

35. Finney, D.J. Probit analysis, Cambridge University Press*.* Cambridge, UK, (1971).
